# Emotional Bookkeeping and High Partner Selectivity Are Necessary for the Emergence of Partner-Specific Reciprocal Affiliation in an Agent-Based Model of Primate Groups

**DOI:** 10.1371/journal.pone.0118921

**Published:** 2015-03-18

**Authors:** Ellen Evers, Han de Vries, Berry M. Spruijt, Elisabeth H. M. Sterck

**Affiliations:** 1 Animal Ecology, Utrecht University, Utrecht, the Netherlands; 2 Ethology Research, Biomedical Primate Research Center, Rijswijk, the Netherlands; Universidad Carlos III de Madrid, SPAIN

## Abstract

Primate affiliative relationships are differentiated, individual-specific and often reciprocal. However, the required cognitive abilities are still under debate. Recently, we introduced the EMO-model, in which two emotional dimensions regulate social behaviour: anxiety-FEAR and satisfaction-LIKE. Emotional bookkeeping is modelled by providing each individual with partner-specific LIKE attitudes in which the emotional experiences of earlier affiliations with others are accumulated. Individuals also possess fixed partner-specific FEAR attitudes, reflecting the stable dominance hierarchy. In this paper, we focus on one key parameter of the model, namely the degree of partner selectivity, i.e. the extent to which individuals rely on their LIKE attitudes when choosing affiliation partners. Studying the effect of partner selectivity on the emergent affiliative relationships, we found that at high selectivity, individuals restricted their affiliative behaviours more to similar-ranking individuals and that reciprocity of affiliation was enhanced. We compared the emotional bookkeeping model with a control model, in which individuals had fixed LIKE attitudes simply based on the (fixed) rank-distance, instead of dynamic LIKE attitudes based on earlier events. Results from the control model were very similar to the emotional bookkeeping model: high selectivity resulted in preference of similar-ranking partners and enhanced reciprocity. However, only in the emotional bookkeeping model did high selectivity result in the emergence of reciprocal affiliative relationships that were highly partner-specific. Moreover, in the emotional bookkeeping model, LIKE attitude predicted affiliative behaviour better than rank-distance, especially at high selectivity. Our model suggests that emotional bookkeeping is a likely candidate mechanism to underlie partner-specific reciprocal affiliation.

## Introduction

Observing social grooming in primates, it quickly becomes clear that grooming does not only serve a hygienic function of removing ectoparasites or dust particles from the other animal’s skin [1,2]. The groomed animal [3,4], and, to a lesser degree, also the grooming one [3,5,6] becomes more relaxed, as indicated by the decrease of observable signs of distress such as self-scratching [4] and heart rate [7,8]. Whether primates can integrate such pleasant grooming episodes in their socio-emotional memory and associate it later with former grooming partners is at present unknown, but not unlikely [9–11]. The general significance of grooming in building and maintaining affiliative relationships in primates is widely accepted [12,13]. The relative strength of an affiliative relationship depends on the frequency, intensity and distribution of grooming and other affiliative interactions.

As many primate studies have shown, grooming behaviour is often reciprocated [14–16]. For the occurrence of reciprocal grooming between two individuals A and B, the following preconditions are often assumed to be necessary: (i) A and B should repeatedly encounter each other, (ii) they should be able to recognize each other, (iii) animal A should be able to memorize grooming it had received from B in order to return these, and (iv) animals should be able to select such grooming partners from which they had received grooming earlier. While there is no doubt about the necessity of the first condition, the necessity of the 3 cognitive abilities is debated (see further below). Group-living allows animals to interact repeatedly; however, encounter frequencies are not random and can even be zero for some dyads. Individual recognition is a cognitive ability possessed by many animal taxa, ranging from insects to primates [17]. Condition (i) and (ii) are, thus, easily met by social primates. Regarding condition (iii), it is unclear what aspects of the quality or quantity of earlier grooming acts should be memorized to allow for reciprocity. Similarly, regarding condition (iv), it is unknown how strict such partner selectivity should be when there are several interaction partners to choose from. In this paper we use a simulation model to explore these two uncertainties to illuminate the cognitive capacities that may be necessary for reciprocation of affiliative behaviour among primates living in a social group.

Diurnal primates are group-living animals that are found to have reciprocal affiliative and supporting relationships [15]. On the basis of variation in the rate of affiliation, proximity and support, social relationships with group members are considered to range from high to low quality [12,18–20]. Individuals with high quality relationships typically exchange affiliative and support behaviour [21]. In primates, the presence of such high quality relationships has been related to different characteristics, such as kinship [22], similarity in dominance rank ([23,24]; but see [25]) and age-similarity ([26], but see [27]). Yet, while these characteristics are often predictive of the quality of a dyadic relationship, they are statistical predictions and appreciable variation in the strength of the social bonds exists among dyads with similar characteristics (kinship: [28,29], dominance rank: [30], age-similarity: [31]; for all three: [32,33]). For instance, the degree of grooming reciprocity varies substantially between dyads of the same dominance rank class in Tibetan macaques (see Fig. 3 in [30]). In baboons [32], the strength of social bonds (measured via a sociality index based on grooming and proximity) between females that are not related via maternal lines is significantly correlated with rank distance. However, the R^2^ is only 0.0243, which means that 97% of the variation in dyadic sociality in the 1,212 dyads is left unexplained by rank distance. Similarly, also age proximity correlated significantly with dyadic sociality (R^2^ = 0.0194, N = 1,212), leaving 98% of the variation in dyadic sociality unexplained. Only for relatedness, either through maternal lines or through maternal and paternal lines, a substantial correlation was found (R^2^ = 0.221, N = 1,416 and R^2^ = 0.311, N = 489, respectively). Still, about 70% of the variation in dyadic sociality is left unexplained by kinship. Apparantly, reciprocal grooming relationships are not only determined by general dyadic characteristics such as kin, rank difference and age proximity, but can also be specific for the partners in the dyad. How this partner-specific reciprocity in affiliative relationships can arise in a social group of primates is one of the main questions addressed in our simulation study.

Although variation in relationship quality is overwhelmingly present in primate groups, researchers disagree whether primates themselves regulate their behaviour on the basis of relationship quality. One line of thought argues that variation in relationship quality emerges from interaction patterns and the relationship of a dyad is an epiphenomenon recognized only by human observers but not by the primates themselves [34–36]. In this view, interactions are the short-term contingent responses to current needs, without memory of previous interactions or anticipation of future ones playing a role [37]. In contrast, others argue that primates know their own relationships with group members and even relationships among others [19,38]. Some memory of past interactions signifies the quality of relationships with others and affects the selection of current interactions with them [39] mediated by current emotions and socio-emotional memory [40]. Complex cognitive capacities, such as anticipation of future needs or interactions, are not required [39]. Consequently, several different underlying cognitive mechanisms have been proposed to regulate reciprocation of affiliative behaviour, ranging from cognitively simple to complex.

A cognitively simple mechanism may suffice to generate reciprocation at a group level when there is a persistent pattern in spatial proximity among the group members [41]. This spatial pattern may result from similarity in individuals such as similarity in dominance rank [42–44]. Here, individual recognition is not required, since the selection of a behaviour towards a group member nearby may only be influenced by the (observable) rank difference between individuals and the degree of tension of the actor, but not by the identity of group members involved in earlier (affiliative) interactions with ego [41]. When individuals use symmetry in a characteristic, such as rank, age, sex or kinship, to direct their behaviour, this is called *symmetry-based reciprocity* [45]. This mechanism does not require animals to keep track of interactions with others in the past, because only the currently perceivable state of affairs determines the behaviour of each animal.

A more complex mechanism involves taking only a recent event into account, in which case one speaks of reciprocity through *short-term temporal contingency* [21] or of *attitudinal reciprocity* [45,46], that is, the individual’s behaviour mirrors the attitude of its partner just before [45,46]. This requires individual recognition, unless the reciprocated behaviour is always executed simultaneously with or immediately after the received behaviour, as is the case in mutual grooming. While originally attitudinal reciprocity was conceived to act only in the short term, later definitions released this restriction [21], by which it became effectively equivalent to the concept of *emotional bookkeeping*. The short-term temporal contingency mechanism and the short-term attitudinal reciprocity mechanism can in fact both be considered as specific cases of emotional bookkeeping, namely when only the most recent beneficial act is remembered for a (very) short while.

Taking the emotional experiences from past interactions with specific partners into account over a longer time span is called *emotional bookkeeping* [19,21,47]. Here, individuals assign and update long-lasting emotional attitudes towards specific partners based on earlier interactions, without remembering these events specifically. This mechanism evidently requires individual recognition. The effects of these long-lasting emotional attitudes are explored in the current paper. The effect of the time span over which emotions elicited by earlier interactions are integrated into these attitudes is explored in another study using the EMO-model [48].

The cognitively most advanced mechanism is *calculated reciprocity* [45], where an individual keeps track of past interactions with different partners and behavioural decisions are based on these memorized interactions such that received favours are equally returned.

Which cognitive process underlies reciprocity is still under debate. However, evidence is mounting that social behaviour is mediated by emotional processes [9,19,20]. Therefore, emotional bookkeeping has been proposed as a candidate mechanism to integrate positive emotions due to affiliative behaviour without requiring high cognitive abilities of remembering exactly who did what to whom and when (i.e. episodic-like memory, [49]). Emotional bookkeeping involves tracking of the current valuation of group members based on previous affiliative interactions. Whether emotional bookkeeping plays a role in reciprocity is difficult to study in real animals, due to the complexity of primate social life, but its effects on patterns in social behaviour can be studied theoretically in an agent-based model.

Besides socio-emotional memory, another factor influencing the emergence of reciprocal affiliative behaviour is partner choice [50]. Partner choice is often discussed in the context of the biological market approach [51]. Depending on the supply and demand of different commodities, such as grooming and tolerance, and their relative values, an individual should select an interaction partner based on the availability of these commodities within the group for *that* individual. This assessment of potential grooming partners may, at least partly, be based on the memory of received affiliation from those individuals so far. For instance, when grooming is assumed to be the only commodity, it is predicted that good reciprocators preferentially groom each other, which was indeed found (see table I in [25]). Thus, application of the biological market approach to altruistic acts such as grooming presupposes that selection of a grooming partner is, at least partly, based on memory of the amount of received grooming from others. How strict this partner selection should be, that is, to what degree A’s decision to groom B rather than C should depend on the socio-emotional memory of grooming received from B and C in order for reciprocity of grooming to arise, is unknown. It is the main question of this modelling study. Agent-based models (ABM) formulate characteristics of interacting entities and study the group-level patterns that emerge from their interactions. Previous ABMs modelling the effect of social interactions among group living animals have investigated group level patterns like dominance relationships and spatial ordering (bumble bees: [52]; primates (macaques): [42]). The behavioural rules in these models regulate behaviour on the basis of relative dominance position (the chance to win or lose a fight). Later versions also included an emotional reaction to aversive events (receiving aggression enhances anxiety) that in turn affect affiliative behavioural tendencies (i.e. grooming; [41,53]). These models generate reciprocal patterns in grooming [41], but do not require individual recognition or emotional bookkeeping. However, real animals do not only entertain negative emotions (anxiety), but also positive ones [54].

A recent ABM by Campenni and Schino [55] focuses on partner choice based on benefits received, without modeling the spatial distance between individuals. In their model, at each step a random agent in the population (N = 50) is selected as actor, after which a random subset of candidates (N = 2, 10, 25 or 49) from the population is extracted. Based on its memory (comprising 0, 5, 100 or 1000 previous steps) of the cooperative interactions received from the candidates, one of these candidates is chosen by the actor as receiver of its cooperative act. The type of cooperative act is unspecified in the model, but may be grooming, tolerance around resources or aggressive coalitions, i.e. what generally is designated as ‘altruistic behaviour’ (cf. [29,47]). In their “single-generation” model they found a correlation across pairs between cooperative acts given and received, and also found considerable differences in frequencies of exchanged cooperation between dyads. Both findings were more pronounced in a setting with a longer time span of memory of received cooperative acts.

To study the effect of both negative and positive emotions on patterns of social behaviour while also modelling the spatial distances among group members we developed the EMO-model [56]. The EMO-model is a first attempt to model the social behaviour of group living primates where the behaviour of the individuals, including distance regulating behaviour such as approach, flee and avoid, is regulated by two different emotional dimensions and the degree of their arousal. We took great care to ground all behavioural and emotional processes implemented in our model on empirical studies. Although we did not succeed fully in doing so, many simulation results corresponded with empirical data [56]. The EMO-model assumes that anxiety and satisfaction are two important emotional dimensions regulating different types of behaviour. The model animals also have an arousal state. A high arousal state increases the probability to perform some active behaviour and also increases the probability to scan the environment for group members.

Besides anxiety and satisfaction, which are influenced by behaviour but are independent of the interaction partner, we also provided each model animal with partner-specific emotional attitudes, i.e. LIKE and FEAR attitudes. The LIKE attitude towards a specific individual is dynamically updated after engaging in affiliative behaviour (i.e. grooming) with that individual. The update depends on the extent of the emotional response, its satisfaction, elicited by receiving or giving grooming, i.e. on the duration and direction of grooming bouts. Without new grooming interactions, LIKE attitudes slowly decrease over time. An update of the LIKE attitude towards animal A integrates the current emotional state (satisfaction) due to current grooming with A with the current emotional memory (LIKE) due to earlier grooming with A. Thus, the individual does not remember the specific grooming events with partner A, but retains one dynamic emotional LIKE attitude towards individual A. In this way, the model animals are provided with an emotional bookkeeping mechanism. Note, that this mechanism is much simpler than episodic(-like) memory or calculated reciprocity (45), because there is no memory of individual events or episodes and their order in time, to be recollected later.

As the current version of the EMO-model focuses on the effect of the dynamical LIKE attitudes, FEAR attitudes were kept fixed and purely dependent on the (fixed) rank distance between individuals. Previous models investigated the dynamics of dominance relations in relation to spatial structure [42]. However, in many primate species, dominance hierarchies can be stable over long periods up to several years (macaques: [57–60], gorillas: [61], baboons: [62,63], capuchins: [64], vervets: [65]) and are altered only incidentally, e.g. after changes in the group composition due to birth, death or migration of individuals [61,66] or destabilizing coalitions [67]. Moreover, similar spatial distribution patterns were found independent from modelling dominance relations as dynamic or fixed [43,68]. Thus, to keep things simple, we decided to model a primate group with a stable dominance hierarchy, where each individual had a fixed dominance strength.

In this paper we will use the EMO-model to investigate how and when affiliative relations a) emerge, b) are self-reinforcing, and c) are maintained. Moreover, we study how reciprocity in affiliative relations depends on one key parameter in the model, i.e. the degree of partner-selectivity determined by the parameter LIKE-Partner Selectivity (LPS, see methods, point 14.). Lastly, we investigate whether emotional bookkeeping is necessary for reciprocal affiliative relationships to emerge. To do this, we study a control model with fixed instead of dynamic LIKE attitudes. In this cognitively simpler *fixed LIKE attitudes model*, both FEAR and LIKE attitudes are based on the rank distance between individuals: symmetry-based reciprocity may arise when individuals simply prefer grooming partners of similar rank. We explore the differences between the reciprocal affiliative relationships that emerge in the *fixed attitudes model* compared to the *dynamic attitudes model*.

## Methods

Simulations were run using NetLogo 5.0.2 [69]. The program code of all models will become available via the *Publications* website of the *Animal Ecology* group (http://www.uu.nl/faculty/science/EN/contact/depts/biology/research/chairs/bb/Publications/Pages/default.aspx). Below, we describe our models according to the updated ODD protocol [70], which is a standardized method of describing agent-based models. This ensures the model description to be more complete and better comparable to other models, allowing also reproducibility of the model. The ODD protocol contains an Overview (1–3), Design concepts (4–11), and Details (12–13). The detailed ODD protocol information on the EMO-model can be found elsewhere [56]. Additionally, this methods section includes parts on the 14) Simulation experiments and the 15) Statistical analysis, which are not part of the ODD protocol.

### 1. Purpose

The purpose of the model described in this paper was to explore certain capacities of information integration that may be used in primates to develop and maintain social relationships, and their effect on the emergent properties of affiliative relationships. While the model was intended (and parameterized) to specifically investigate social behavior of macaques, we believe that general model processes (such as the emotional regulation of behavior and emotional bookkeeping) may be very similar across many primate species. The resulting group level patterns may also apply to other New World or Old World monkeys and apes, and possibly even to prosimians. We studied affiliative partner selectivity, i.e. the degree of preferring certain individuals as affiliation partners. Moreover, we compared the use of dynamic affiliative attitudes based on partner-specific affiliation history and emotional bookkeeping to fixed affiliative attitudes simply based on rank-distance.

### 2. Entities, State Variables and Scales

We modelled the movements and interactions of 20 individuals. The individuals are characterized by a number of state variables (Table T1 in S2 Supplementary Material and see below). These state variables are identical to the ones used in the introductory paper on the EMO-model [56], with the exception of fixed LIKE attitudes we use here in a control model.

Individuals are characterized by their dominance strength (myDOM), which does not change over time or after interactions [43,44,56,68]. Individuals differ in their schedule time (myTIME), (current) scanning probability (myPscan) and in the current width of the view angle (myVIEW_ANGLE), which change dynamically over the course of the simulation.

Our model entities are further described by their emotional state, consisting of arousal, anxiety and satisfaction (myAROUSAL, myANXIETY and mySATISFACTION), i.e. an individual’s state of alertness and an aversive and a pleasant dimension of the emotional state. Arousal, anxiety and satisfaction may change dynamically over time depending on the social context ego (i.e. a model entity) had experienced. The level of arousal, anxiety and satisfaction that is approached over time is described by the respective limit value (myAROUSAL_LIMIT, myANXIETY_LIMIT, mySATISFACTION_LIMIT). Model individuals are also characterized by partner-specific emotional attitudes (LIKE and FEAR), they assign to each other group member. In our model FEAR attitudes are fixed, while LIKE attitudes are dynamically changing over time depending on earlier affiliative interactions. Only in a control model, the fixed attitude model, LIKE attitudes were fixed.

General model parameters are identical to those used in the introductory paper on the EMO-model [56] and are summarized in table S2 in [56]. The modelled environment is a continuous two-dimensional grid (300 × 300 grid units) with a torus shape to exclude disturbing border effects. The length of one grid unit resembles 1 “meter”. We did not explicitly implement ecological features of the environment; in the model an individual’s environment is purely social.

One time step in the simulation resembles 1 MINUTE. One HOUR consists of 60 MINUTES and we defined 12 HOURS as one DAY, 7 DAYS as 1 WEEK and 50 WEEKS as 1 YEAR. Simulations were run for 504.000 time steps, i.e. 2 YEARS, plus a prior stabilization period of 21.600 time steps, i.e. ca. 4 WEEKS.

### 3. Process Overview and Scheduling

Our model is event-driven. Most social behaviours are modelled as discrete events in time, except for moving, resting and grooming, which are modelled as continuous duration behaviours. Time is modelled on a continuous scale and during a simulation run individuals’ activations are regulated by a timing regime. The general process overview and the scheduling of individuals to start a new behaviour are identical to those described in the introductory paper on the EMO-model [56] (Figure F1 in S1 Supplementary Material). The reader is referred to this paper.

### 4. Basic Principles

In our model, given, received and observed behaviours affect the general emotional state (arousal, anxiety and satisfaction) of individuals. In turn, an individual’s emotional state affects its general short-term probability of executing certain behaviours. In this way, emotions regulate spontaneous behaviours as well as appropriate responses to received behaviours. More specifically, receiving and executing affiliative behaviour increases satisfaction levels and decreases arousal and anxiety. While receiving submissive behaviours results in decreased arousal and anxiety, receiving aggressive behaviour or perceiving aggression nearby results in increased levels of anxiety and arousal. Executing aggressive behaviour increases ego's own arousal, while ego's own anxiety level decreases or increases depending on the outcome of the conflict. In turn, high arousal levels result in a higher general probability for all active behaviours, high satisfaction levels decrease the probability of (further) affiliation and high anxiety levels result in increased probabilities for affiliative or submissive behaviour and in more risk-sensitive aggression probabilities.

In the dynamic attitude model, partner-specific LIKE attitudes summarize earlier received affiliation from specific individuals on a longer-term. More specifically, receiving affiliation from a specific individual increases ego's LIKE attitude towards this individual. In turn, ego's probability to affiliate with this specific individual increases. In a control model, the fixed attitude model, the feedback regulation between affiliation and LIKE attitudes is absent and LIKE attitudes are fixed, i.e. they depend on similarity of dominance and are not affected by behaviour.

In sum, the emotional state regulates appropriate behaviour in response to received behaviours, while partner-specific emotional attitudes regulate appropriate behaviour in response to specific individuals. A more detailed description of the regulation of behaviour via emotions and dynamic LIKE attitudes can be found elsewhere [56].

### 5. Emergence

In agent-based models, individual behaviour is imposed by the model rules, while group-level properties are usually not implemented explicitly into the model, but rather emerge from the interactions of the lower-level entities, i.e. the individuals. In our model, behavioural patterns, e.g. affiliation, aggression and proximity, are emergent properties arising from the interactions of the model entities. The structure of the network of LIKE attitudes and group level properties such as reciprocity and partner-specificity are an emergent property arising from the interrelation between emotional attitudes and affiliative behavior.

### 6. Adaptation

The model entities change their behaviour in response to changes in their general emotional state and their emotional attitudes towards others and individuals (implicitly) seek to increase satisfaction and to decrease anxiety. As appropriate behaviour is mediated by emotional processes this yields a homeostatic regulation system. In this way, we aimed to produce adaptive (in the sense of flexible) behaviour and emerging group properties that are representative of observations of the social behaviour of real primates.

### 7. Learning

Individuals in the dynamic attitude model regularly update their partner-specific LIKE attitudes assigned to other group members, based on earlier grooming received from these individuals. This may be seen as a (basic) form of 'learning'. Emotional bookkeeping provides individuals with summarized information on 'valuable' affiliation partners, which may dynamically change over time according to these partners' behaviour. In this way, individuals 'learn' with which specific partners they should affiliate.

### 8. Sensing

The individuals' sensing capabilities in this model are identical to those used in the introductory paper on the EMO-model [56]. Individuals in our model may perceive the location, certain behaviours and signals of other group members, but only 'locally' within certain distances and within a specific view angle. The exact distances depend on the salience of the perceivable information. Individuals are able to perceive (or know) the dominance strength of other group members and perception of a group member elicits ego's internal valuation of this group member, i.e. its FEAR and LIKE attitude that are assigned to this specific individual.

### 9. Interactions

Interactions are implemented identically as described in the introductory paper on the EMO-model [56]. Social interactions in our model can be categorized as affiliative, submissive and aggressive behaviours. Affiliation comprises grooming, affiliative signalling and approaching; submission comprises leaving, submissive signalling and avoiding; and aggression comprises attacking and aggressive signalling.

Potential interaction partners are the 10 nearest recognizable individuals (within MAX_DIST and ego's current view angle). The potential behavioural probabilities towards these 10 (or less) individuals depend on ego's emotional attitudes (FEAR and LIKE) assigned to those individuals and ego’s general emotional state (arousal, anxiety, satisfaction). One behaviour towards one specific interaction partner is chosen randomly according to these probabilities.

Some social interactions can only be performed towards group members within a certain distance. Individuals within INTERACT_DIST (1m) can be groomed, left or attacked. Individuals within PERS_DIST (5m) can receive (affiliative, submissive or aggressive) signals. Individuals within MAX_DIST (50m) can be approached and individuals within PERS_DIST (5m) can be avoided.

### 10. Stochasticity

In our model, many processes are not implemented deterministically, but include some degree of stochasticity, to produce variability in those processes. Those processes include action selection, the determination of the winner of an escalated fight, the random walk procedure and the timing regime.

### 11. Observation

For the analysis of our model, we only used data that were recorded during the last 252.000 time steps of each simulation run, i.e. the last YEAR.

The individuals' levels of arousal, anxiety and satisfaction, the dyadic proximity scores and the levels of dyadic LIKE attitudes were sampled every 3.5 DAYS and then averaged (per individual or dyad, respectively) over one YEAR for each simulation run. The number or duration of dyadic behaviours was recorded per dyad per behaviour over each recording interval of 3.5 DAYS and then divided by the duration of the recording interval to obtain average hourly behavioural rates.

To assess the average dyadic proximity score, i.e. the average rate of being located in each other's proximity, we scored for each individual which other group members were found within close proximity (1m) at the time of sampling using the “one–zero” sampling technique. Thus per dyad, possible scores were 1 (in proximity) or 0 (not in proximity) per sample. Note, that proximity is by definition a symmetric measure, while LIKE attitudes and social interactions are always directed from an actor to a receiver and are thus not symmetric by definition. Per individual this translates into possible scores between 0 (no other group member was in proximity) and 19 (all other group members were in proximity).

### 12. Initialization

The initial settings are summarized in Table T1 in S2 Supplementary Material and are identical to the introductory paper on the EMO-model [56], with the exception of fixed LIKE attitudes in the control model. At the initialization of each simulation run, the x-coordinates and the y-coordinates of the 20 individuals were drawn randomly from a predefined circular sphere with an arbitrary diameter of 50m. The individual's initial heading was set to a random orientation between 1º and 360º and the initial view angle was set at 120º for each individual. Each individual's level of arousal was set to the default arousal level (0.09) and the level of anxiety and satisfaction was set to 0.0. In the dynamic attitude model, LIKE attitudes were initialized at 0.0. In the control model, the fixed attitude model, LIKE attitudes were fixed and calculated as a function of (absolute) rank-distance: LIKE_ij_ = max(0, 0.243 — abs(myDOM_j_—myDOM_i_)*0.36). The initial schedule time for each individual was drawn randomly from a normal distribution with a mean of 1 minute and a standard deviation of 0.05 minutes.

### 13. Submodels

The implementation of all processes in our model, except for the fixed LIKE attitudes in the control model, is identical to the introductory paper on the EMO-model [56]. This section broadly describes the main processes implemented in the EMO-model. It covers the implementation of emotional state (arousal, anxiety and satisfaction), partner-specific emotional attitudes (LIKE and FEAR), action selection, perception, scanning, movement, grouping, grooming and resting, and attack, counter-attack and escalated fight. For a detailed description of the model, the substantiation of the modelled emotional processes and behavioural rules using empirical data, and parameterization and validation of our model we refer the reader to our earlier paper [56].

#### Arousal

In our model, an individual's arousal level, i.e. its responsiveness or tendency to be active, increases in response to receiving, executing or observing aggression or when in proximity of a dominant individual, i.e. within 5m (PERS_DIST). On the other hand, arousal may decrease over time and in response to receiving submissive or affiliative behaviour and executing affiliative behaviour. The extent of arousal change depends on the emotional salience of the stimulus (Table T3 in S2 Supplementary Material). Arousal level was scaled between 0 (inactive) and 1 (highly stimulated), with 0.09 being the baseline level (DEF_AR_LIMIT). Higher arousal was implemented to result in an increased probability of performing active behaviours (any behaviour except resting) and in an increased probability to employ social vigilance, i.e. scanning behaviour.

#### Anxiety

In our model, anxiety level, i.e. an individual's general 'fearfulness' in response to negative stimuli within the current social environment, was scaled between 0 (not anxious) and 1 (highly anxious). Anxiety increases over time and in response to aggression, i.e. 'Receiving or Giving an attack', 'Receiving an aggressive signal', 'Losing an (escalated) fight' or 'Observing an escalated fight nearby', and decreases in response to successful aggression, submission and affiliation, i.e. 'Winning an (escalated) fight', 'Receiving a submissive or affiliative signal' and 'Receiving or Giving grooming'. The extent of anxiety change depends on the emotional salience of the stimulus (Table T3 in S2 Supplementary Material). High anxiety levels generally result in increased probabilities of affiliation and submission and decreased probabilities of aggression.

#### Satisfaction

In our model, satisfaction level, i.e. an individual's general 'contentedness' in response to positive stimuli was scaled between 0 (not satisfied) and 1 (highly satisfied). Whenever two animals engage in grooming, current satisfaction levels increase with linear rates, namely 0.05/min for the groomer and 0.1/min for the groomed individual. Whenever grooming had stopped, satisfaction decreases again to baseline level with a default linear decrease rate of 0.02/min, i.e. within one hour. (Table T3 in S2 Supplementary Material). High satisfaction results in decreased probabilities of affiliation.

#### FEAR Attitudes

In our model, individuals assign a partner-specific FEAR attitude to each group member. FEAR attitudes resemble the difference in dominance strength between an individual (i) and another group member (j) and are calculated as FEARij = myDOMj-myDOMi, i.e. ranging from -0.95 to +0.95. Thus, FEAR attitudes are directional and not symmetric. FEAR attitudes are fixed over the course of our simulation and are, thus, not affected by social interactions. Yet, they do affect the individual's valuation of its potential aggression risk related to the respective group member. High FEAR attitudes result in decreased probabilities of aggression (i.e. attack, aggressive signal) and increased probabilities of submission (i.e. leaving, submissive signal, avoidance) towards the respective group member.

#### LIKE Attitudes

LIKE_ij_ describes an individual's (i) affiliative valuation of a specific group member (j). LIKE attitudes may have values ranging from 0.0 (neutrally valued affiliation partner) to +1.0 (highly valued affiliation partner). In both the dynamic and fixed attitude model, a higher LIKE attitude towards a group member implies, modified by the value of the LIKE-Partner Selectivity (LPS, see below), higher probabilities of affiliation (i.e. grooming, affiliative signalling and approaching) towards this group member.

In the control model (fixed attitude model) LIKE attitudes are reversely related to the (absolute) rank-distance of two individuals and calculated as LIKE_ij_ = max(0, L_0_-L_S_*abs(myDOM_j_—myDOM_i_)) Here, L_0_ and L_S_ are fixed conversion parameters set to L_0_ = 0.243 and L_S_ = 0.36, to result in a distribution of LIKE attitudes similar to the one emerging in the dynamic attitude model. In the fixed attitude model, by definition LIKE attitudes are symmetric within a dyad.

In contrast, in the dynamic attitude model, LIKE attitudes are not symmetric per se, as they are dynamically updated upon receiving grooming. The exact increase of LIKE_ij_ depends on individual i's current increase in satisfaction in response to grooming received exclusively from individual j, described by the partner-specific variable PARTNER_SAT_ij_, which increases and decreases with the same rate as the general satisfaction level (GR_SAT_INC). Partner-specific LIKE attitudes are then used to integrate earlier affiliation received from a partner, i.e. the changing level of PARTNER_SAT_ij_ over time as follows:

LIKEij(tn)=max{LHW∗LIKEij(tn−1)+(tn−tn−1)∗PARTER_SETij(tn)LHW+(tn−tn−1)PARTNER_SATij(tn)}

Here, t_n_ is the current time, t_n-1_ is the time of the last update and (t_n_-t_n-1_) is the time since the last update (in MINUTES). LIKE_ij_(t_n_) is the updated value of the LIKE attitude assigned from individual i to j and LIKEij(t_n-1_) is the former level of LIKE to be updated. LHW (LIKE-HISTORY WEIGHT) is a fixed parameter (arbitrarily set to 1 DAY), which causes (emotional responses to) earlier affiliation history to weigh stronger than (emotional responses to) affiliation received recently (within the last few MINUTES). While current affiliation received from a partner may quickly increase LIKE and/or maintain a high LIKE, the lack of current affiliation will result in a slowly decreasing LIKE attitude. See [56] for a more detailed explanation of the implementation of LIKE attitudes in the model.

#### Action Selection

In our model, activated individuals may select one of various possible actions. This action may be directed to another individual or may be resting or random movement within the group. Action selection comprises the random choice of a behaviour out of a set of behaviours according to the current probabilities assigned to these behaviours. The probability for a specific behaviour directed towards another group member depends on a) the distance of the individual to ego, b) ego's emotional state (arousal, anxiety and satisfaction), c) ego's FEAR and LIKE attitudes assigned to the potential interaction partners and d) the parameter setting of LIKE-PARTNER SELECTIVITY (LPS), i.e. the degree to which high LIKE attitudes are important for the selection of affiliation partners. The emotional state facilitates behavior that is appropriate to the individual's position and situation within the social group in general, while emotional attitudes facilitate behavior that is appropriate towards specific group members. For a detailed explanation of how the behavioural probabilities depend on the emotional state and the LIKE and FEAR attitudes as well as the LIKE-Partner Selectivity see [56] (especially therein: Fig. 3 and supplementary text S2 for the exact calculation of the probabilities).

#### Perception

The perception module in our model defines the viewing capacities of the agents, which are explained in detail in [56]. Individuals in our model can individually recognize other group members within a maximum perceivable distance of 50m (MAX_DIST) and within the currently employed view angle. The view angle is by default 120º (VIEW_ANGLE) or else 360º (MAX_ANGLE) when ego is scanning. Model entities can judge whether at least three other group members are present within 20m (NEAR_DIST) and within the currently employed view angle. Furthermore, individuals in our model are capable to judge whether their distance to the furthest group member exceeds 100m (FAR_DIST). The two latter criteria are used by ego to decide whether grouping behaviour should be executed. Individuals can also perceive aggressive, submissive and affiliative signals directed at them by others from within 5m (PERS_DIST).

#### Scanning

When employing scanning behavior, an individual is turning its head right and left, thus expanding its view angle to 360º (MAX_ANGLE) instead of the default view angle of 120º (VIEW_ANGLE). The probability to perform scanning behaviour increases with ego's current arousal level [56].

#### Movement

Concerning movement behaviour, individuals in our model may either move from or towards other group members (approaching, grouping, fleeing, leaving and avoiding) or they may execute random movement within the group. Movement behaviour in our model takes time and is implemented as movement bouts. During such a movement bout, movement is executed step by step. After starting a movement bout, ego is activated each 3 SECONDS to execute the movement it was to perform during this time interval and to decide whether movement is to be continued.

After ending a movement bout ego always performs a proximity update. Ego checks whether any individuals towards which it directs a (high) FEAR attitude (i.e. higher-ranking group members) are now (or still) nearer than 5m (PERS_DIST), as this has consequences for the level that ego's arousal will approach over time (myAROUSAL_LIMIT). Additionally, other individuals who direct (high) FEAR attitudes towards ego are updated on ego’s new spatial location, which may in turn affect the level of their arousal.

#### Grouping

Before selecting a social behavior, model entities always check whether grouping should be executed. Grouping will be selected if less than three (MIN_OTHERS) group members are located within 20 m (GROUP_DIST) and 360º (MAX_ANGLE) or whenever any group member is further away from ego than 100 m (FAR_DIST). When grouping is to be performed, ego simply approaches any randomly selected group member.

#### Grooming and resting

In our model, grooming and resting behaviour are implemented as duration behaviours, which are executed in bouts. When starting a grooming or resting bout, ego's next activation is scheduled several minutes later to choose its new behaviour. Ego may be disturbed and activated earlier in response to receiving an attack or an aggressive signal, or after observing an escalated fight nearby.

#### Attack, counter-attack and ecalated fight

Upon receiving an attack the respective model individual is immediately activated to respond with either fleeing or a counter-attack. When a counter-attack was selected in response to an attack, we call this an escalated fight. The winner and loser of such a fight are determined randomly according to the individuals' win chances which are calculated via a sigmoidal function of the difference in dominance strength of the two individuals, [43,71]. When no counter-attack is executed, the attacked individual is defined as the loser and the attacker as the winner of this aggressive interaction. After an attack or an escalated fight, the loser flees from the winner, while the winner is scheduled anew shortly after. Whenever an escalated fight takes place, individuals nearby get activated and their arousal level gets increased. Moreover, these individuals are activated shortly after to enable an appropriate reaction in response to the event.

### 14. Simulation Experiments

In this paper we present the results of two different types of our model. First, we describe the *dynamic attitude model* (emotional bookkeeping model), in which individuals dynamically update their LIKE attitudes according to earlier received affiliation by specific group members and subsequently use these LIKE attitudes to choose affiliation partners. Second, we describe the *fixed attitude model* (symmetry-based model), in which LIKE attitudes are also used to choose affiliation partners, but where LIKE attitudes are symmetric and only dependent on rank-distance and thus are fixed. In the fixed attitude model, there is no feedback from affiliative behaviour onto LIKE attitudes. Therefore, the fixed attitude model serves as a control model to assess the importance of this dynamic feedback from behaviour to emotional attitudes for the emergence of certain group patterns.

In the dynamic and the fixed attitude model, we varied the parameter LIKE-PARTNER SELECTIVITY (LPS), which describes the degree to which individuals prefer to selectively affiliate with group members towards whom they assign a high LIKE attitude. LPS was set to 0.0, 0.5, 0.9, 0.95 or 0.99. All individuals in the group were given the same LPS, although in reality it is likely that LPS varies between individuals. It is outside the scope of this paper to explore the effect of different settings of within-group variability of LPS. LPS = 0.0 resembles a special null model setting. Here, individuals have no preference to select specific affiliative partners concerning LIKE attitudes whatsoever. In other words, individuals do not use LIKE attitudes during affiliative partner selection. Therefore, the null model setting of the dynamic and the fixed model are identical. The null model setting serves as a control setting to assess the effect of the presence of any affiliative partner preference based on emotional bookkeeping.

For each setting of LPS, 10 independent simulations were run for the dynamic and the fixed attitude model, resulting in a total of 100 independent simulation runs.

### 15. Statistical Analysis

We first explain how specific summarized measures were calculated from the recorded data, e.g. how data on dyad level were transformed to obtain measures on individual or (sub-)group level. We continue with the statistics that were used to compare the properties of different subgroups or individual categories. Finally, we explain how we calculated specific group properties, i.e. Shannon index, group-level reciprocity, partner-specificity and predictability. All statistical analyses were performed in R 2.15.2 [72].

Individual proximity scores, strength of LIKE attitudes and behavioural rates were calculated as the sum of all dyadic proximity scores, LIKE attitudes or behavioural rates that an individual directed to others. Group means of behavioural rates were calculated as the mean of all individual behavioural rates. To calculate the means per rank category, we divided the 20 group members into 10 lower-ranking (subordinates) and 10 higher-ranking (dominants) individuals and averaged the respective individual proximity scores, strength of LIKE attitudes and behavioural rates per subgroup. To calculate the dyadic means per rank-distance category, we divided all dyads into two similar-sized groups. Dyads for which the absolute difference in dominance strength was less than 0.35 were defined as similar-ranking dyads (N = 99 for symmetric measures and N = 198 for directed measures). Dyads for which the difference in dominance strength was more than or equal to 0.35 were defined as distant-ranking dyads (N = 91 for symmetric measures and N = 182 for directed measures). We then averaged the respective dyadic proximity scores, strength of LIKE attitudes and behavioural rates per subgroup.

To assess how evenly individuals distributed their proximity, LIKE attitudes and social behaviours among all potential partners, we calculated the Shannon index (H). This diversity measure has frequently been used in earlier primate research (e.g. [28,73]). H of individual i was calculated as: *H*_*i*_ = –∑_*j*_ *p*_*ij*_ log *p*_*ij*_ where i is the actor, j are all potential receivers and p_ij_ is the relative proportion of a behaviour given by the actor i to the jth receiver. We calculated H using the yearly averages of the dyadic measures. To compensate for group size, an evenness index was applied to the Shannon index following Buzas & Gibson [74] as: *H*_i_^*^ = exp(*H*_*i*_)/(*N*—1), where N is the group size. H* describes whether a behaviour is directed equally often to all possible partners (H* = 1) or only restricted to one partner (H* approaches 0). H* was calculated per individual and then averaged over the group.

To assess the reciprocity of behaviors and LIKE attitudes at the group level we calculated the Kendall’s tau row-wise matrix correlation between the dyadic interaction matrix (or the LIKE matrix) and its transposed matrix [75,76] using the R software package DyaDA [77].

To assess the variation of dyadic measures within dyads of the same rank-distance, we calculated the standard deviation (SD) of the yearly averages of the dyadic measures. We excluded the dyads of rank-distance -0.95 and 0.95, as they were the only dyads with this rank-distance, which did not allow for calculation of SD. The SDs per rank-distance were then averaged over the group.

To investigate how well either absolute rank-distance or LIKE attitudes corresponded to the behavioural patterns (including also the proximity scores), we calculated the row-wise Pearson correlation coefficients [76] between behavioural patterns and either rank-distances or LIKE attitudes, respectively. To do this we used the yearly averages of these dyadic measures. We calculated the correlation coefficients per simulation run and then averaged them over all runs using a Fisher-z-transformation.

## Results

We present the results of two different types of our model. We describe the *dynamic attitude model* (emotional bookkeeping model), in which individuals dynamically update their LIKE attitudes according to earlier received affiliation from specific group members and subsequently use these LIKE attitudes to choose affiliation partners. Then we compare the dynamic attitude model to the *fixed attitude model* (symmetry-based model), in which LIKE attitudes are also used to choose affiliation partners, but where LIKE attitudes are fixed, symmetric and strictly dependent on rank-distance.

In both the dynamic and the fixed attitude model, we varied the LPS parameter, i.e. LIKE-PARTNER SELECTIVITY, which describes the degree to which individuals prefer to selectively affiliate with group members towards which they direct a high LIKE attitude. LPS = 0 describes the null model setting. In this setting, LIKE attitudes have no effect on affiliative partner selection. Therefore, the null model setting of the dynamic and the fixed model yield the same behavioural patterns.

### 1. Dynamic Attitude Model

In this section we present the results concerning reciprocity, affiliative partner selectivity and the differentiation between rank-distance categories (similar-ranking and distant-ranking dyads).

**Fig 1:**
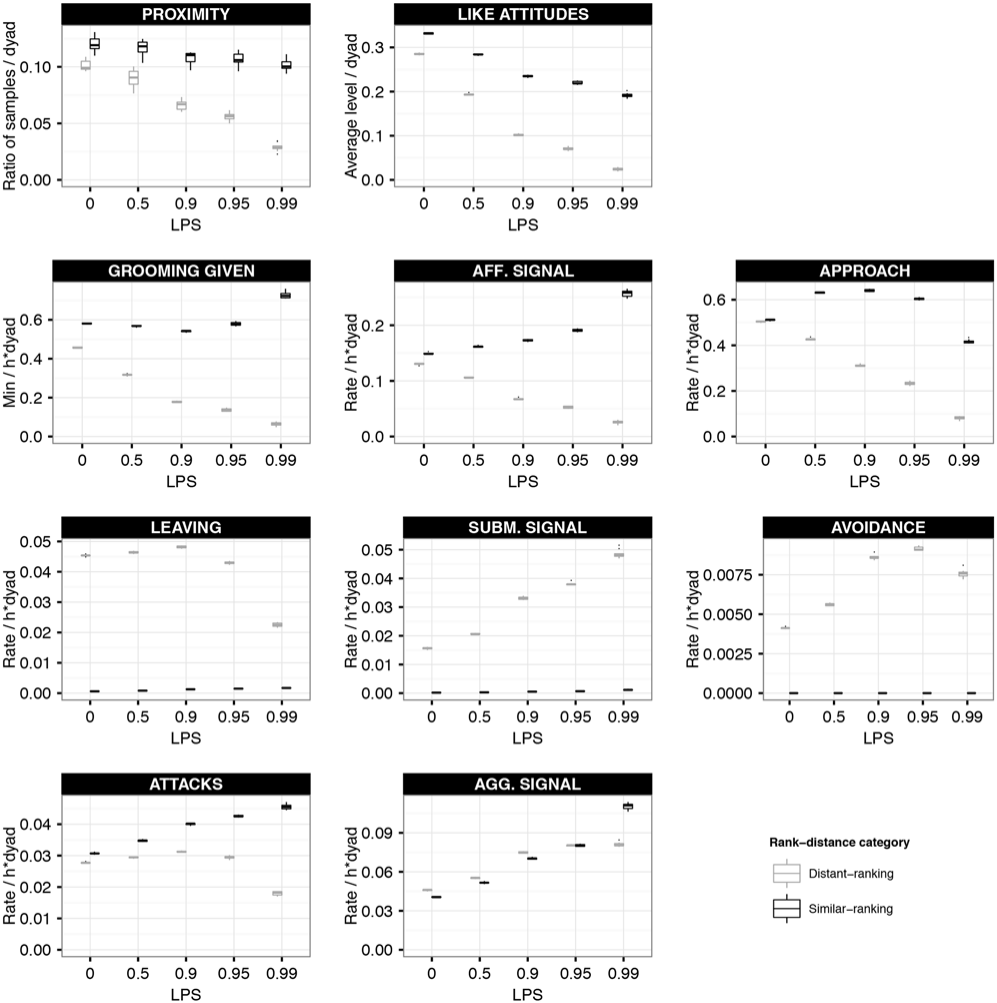
Behavioural rates per rank-distance category in the dynamic attitude model. This figure shows the averaged behavioural rates for distant-ranked (grey box-plots) and similar-ranked (black box-plots) dyads at different settings of selectivity (LPS) in the dynamic attitude model. Note that the setting LPS = 0 refers to the null model setting. Proximity is measured as the ratio of samples in which the members of a dyad were observed in proximity. The level of LIKE attitudes was measured as the average level of all dyadic LIKE attitudes directed to (distant- or similar-ranking) group members. Grooming given is measured in MINUTES per HOUR per dyad. Signals, approach, leaving, avoid and attacks are measured in occurrences per hour per dyad. The box-plots show the results of 10 simulation runs, averaged over 1 YEAR.

**Enhanced Preference for similar Rank at increased LIKE-Partner Selectivity.** We first examined how the behavioural patterns differed between similar-ranked and distant-ranked dyads in the dynamic attitude model. We describe these differences for the null model setting (LPS = 0) and assess the effect of increased LPS (Fig. 1).

Individuals directed grooming and affiliative signals more often to similar-ranking than to distant-ranking group members independently of the setting of LPS. At increased LPS, rates of grooming and affiliative signals generally increased between similar-ranked dyads, while they decreased between distant-ranked dyads. Thus, the preference to affiliate with similar-ranking partners, which was already present at the null model setting (LPS = 0), was reinforced at increased LPS.

The preference to affiliate with similar-ranking group members in turn resulted in higher LIKE attitudes between similar-ranking dyads than between distant-ranked dyads. At increased LPS, values of LIKE attitudes were generally decreased, but they decreased more between distant-ranked dyads than between similar-ranked dyads. This differentiation of LIKE attitudes between similar-ranking and distant-ranking dyads at increased LPS in turn also reinforced the differences in affiliation rates, approach rates and proximity between dyads of different rank-distance categories. At LPS = 0, similar-ranking dyads had slightly higher proximity scores than distant-ranking dyads. Proximity scores generally decreased at increased LPS, but they decreased more between distant-ranked dyads than between similar-ranked dyads.

**Fig 2:**
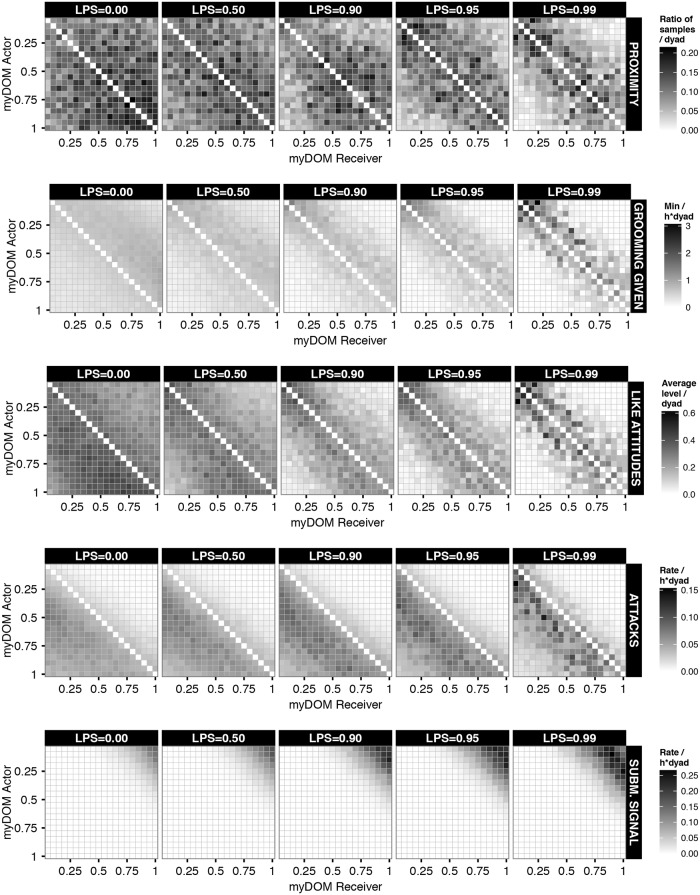
Interaction matrices of behaviours in the dynamic attitude model. This figure shows the dyadic behavioural rates of a group at different settings of selectivity (LPS) in the dynamic attitude model. Behaviours are directed from actors (y-axis) to receivers (x-axis), both are ordered by dominance strength, ranging from low-ranking (myDOM = 0.05) to high-ranking (myDOM = 1.0) individuals. Proximity is measured as the ratio of samples in which a dyad was observed in proximity. Grooming given is measured in MINUTES per HOUR. LIKE attitudes are measured as the average level of an individual's LIKE attitude directed to another group member. Attacks and submissive signals are measured in occurrences per HOUR. The plot shows the behavioural rates of one example run averaged over one YEAR. Dark shades represent high rates or values. Values at the diagonal are by definition not applicable. Affiliative signals and approach are distributed very similarly to grooming given, aggressive signals are distributed very similarly to attacks, avoidance and leaving are distributed very similarly to submissive signals; these patterns are presented in Figure F3 in S1 Supplementary Material.

At increased LPS, the Shannon index of the LIKE attitudes, the proximity scores and affiliative behavioural frequencies were decreased (see black box-plots in Figure F2 in S1 Supplementary Material). Hence, at increased LPS, individuals restricted their affiliative behaviours, their proximity and high LIKE attitudes to fewer group members than at lower LPS. This decrease in number of selected interaction partners explains the slight decrease in proximity scores and LIKE attitudes at high LPS. Averaged over the whole group of similar-ranking dyads these values decrease, while they still increase for those few dyads that mutually prefer each other most (see also Fig. 2 and Figure F3 in S1 Supplementary Material).

At LPS = 0, approach rates were very similar for distant-ranking and similar-ranking dyads. At increased LPS, approach rates were always lower between distant-ranked dyads than between similar-ranked dyads and, similarly to proximity, affiliation and LIKE attitudes, approach rates of distant-ranking dyads decreased. On the other hand, approach rates of similar-ranking dyads first increased at increased LPS (LPS<0.9), as individuals focussed more on indivduals with high LIKE attitudes, which were usually similar-ranking group members. At even higher LPS (LPS>0.9) approach rates of similar-ranking individuals decreased again, as individuals became even more selective and focussed on a few affiliative partners (see above).

Attacks were always more frequent between similar-ranking dyads than between distant-ranked dyads, independently of LPS. At increased LPS, attack rates increased between similar-ranked dyads. As individuals at increased LPS are more often in proximity of similar- than distant-ranking group members, probabilities for attacks are also increased. First, probabilities for aggression directed up the hierarchy are higher when opponents are of similar rank compared to opponents of distant rank. Second, probabilities for counter-aggression are higher when opponents are of similar rank compared to opponents of distant rank (see also Fig. 2 and Figure F3 in S1 Supplementary Material).

Rates of attacks generally decreased between distant-ranked dyads at increased LPS. In our model, attacking another individual is by definition only possible when this individual was in close proximity. As proximity scores between distant-ranked dyads decreased at increased LPS, there are simply less opportunities to direct aggression to distant-ranked individuals.

On the other hand, rates of aggressive signalling, which required only near (5m) and no close (1m) proximity, were very similar between distant- and similar-ranked dyads. Only at highest LPS (LPS = 0.99), similar-ranked dyads used aggressive signals more often than distant-ranked dyads. This may be a result of the fact that at this setting, the relative difference in proximity scores was highest between similar-ranking and distant-ranking dyads and now affected behaviours within not only close, but also near proximity.

At increased LPS, the Shannon index of aggressive behavioural frequencies was only slightly decreased (see black box-plots in Figure F2 in S1 Supplementary Material). Hence, at increased LPS, individuals restricted their aggressive behaviours to slightly fewer group members than at lower LPS, due to their restricted encounters with group members.

Submissive behaviours (leaving, submissive signal, avoidance) were almost exclusively employed (by subordinates) in distant-ranked dyads, independent of the setting of LPS. At increased LPS, rates of leaving, i.e. moving away from someone in close proximity, were generally decreased, simply because individuals spent less time in close proximity of distant-ranking individuals.

At high LPS, rates of submissive signals generally increased. This can be explained as follows. Submissive signals are by definition almost exclusively employed by subordinates, and at low LPS, subordinates may direct either affiliative or submissive signals towards distant-ranking dominants within a certain distance. At increased LPS, affiliative signals are more and more restricted to LIKEd individuals, which are usually similar-ranking. Thus, as the chance of directing affiliative signals towards distant-ranking individuals decreased at increased LPS, the relative chance to direct submissive signals towards these LIKEd individuals increased. Similarly, at low LPS, subordinates may either approach or avoid distant-ranked individuals. As the chance of approaching distant-ranked group members decreased at higher LPS (see above), the relative chance to avoid such individuals generally increased.

At increased LPS, the Shannon index of submissive behaviours showed a minor increase (see black box-plots in Figure F2 in S1 Supplementary Material). Hence, at increased LPS, individuals directed submissive behaviours to slightly more group members than at lower LPS (see also Fig. 2 and Figure F3 in S1 Supplementary Material).

To summarize, the preference for similar-ranking individuals that was already present in the null model setting (LPS = 0) was reinforced at increased LPS due to the mutual positive feedback between affiliative behaviour and the LIKE attitudes. Higher LIKE attitudes towards similar-ranking individuals resulted in higher preferences to approach and affiliate with these individuals, which in turn resulted in the maintenance of high LIKE attitudes towards similar-, but not distant-ranking individuals. In this way, higher LPS resulted in a stronger differentiation between similar- and distant-ranking dyads, with similar-ranking dyads engaging in more affiliation and aggression than distant-ranking dyads. The more LPS is increased, the more individuals restrict their affiliation, prosimity and aggression towards a few preferred partners (Figure F2 in S1 Supplementary Material). The stronger differentiation between similar and distant-ranking dyads at increased LPS is also apparent from the average dyadic values of the behavioural frequencies, proximities and LIKE attitudes (Fig. 2 and Figure F3 in S1 Supplementary Material).

**Fig 3:**
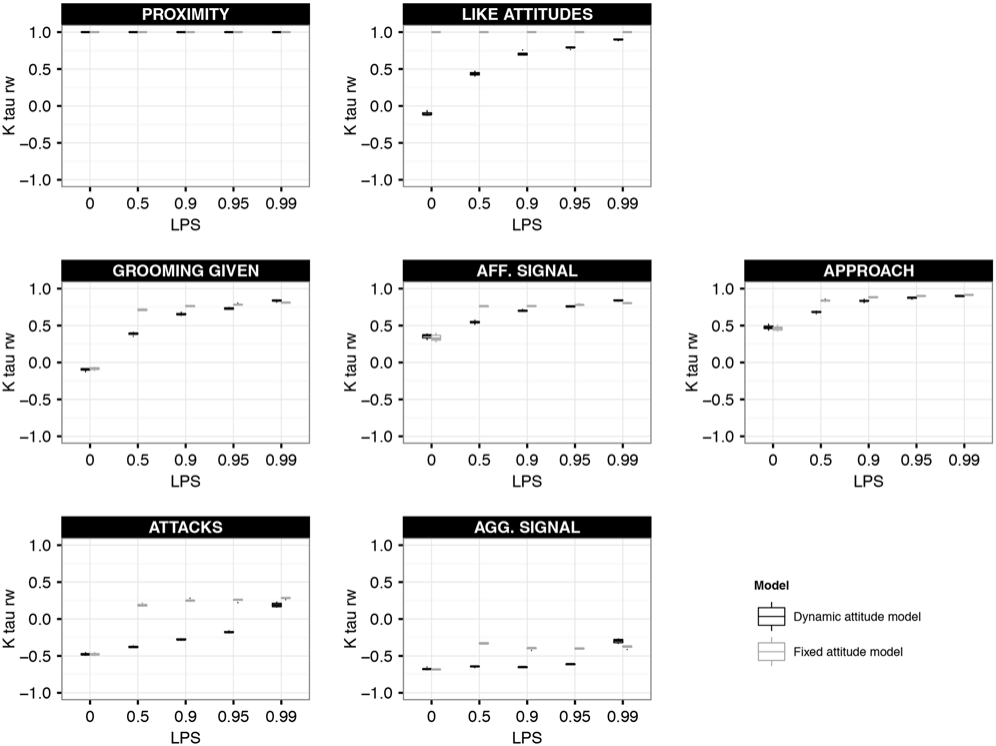
Reciprocity of behaviours. This figure shows the group-level reciprocity of behaviours at different settings of selectivity (LPS) in the dynamic (black box-plots) and the fixed (grey box-plots) attitude model. Group-level reciprocity is measured as Kendall rowwise tau. Positive rowise tau values indicate that behaviours are reciprocated. Negative rowise tau values indicate that behaviours are imbalanced. Rowwise tau values were calculated based on behaviours averaged over one YEAR. The box-plots show the rowwise tau values of 10 simulation runs.

**Enhanced Reciprocity at increased LIKE-Partner Selectivity.** To examine the effect of LPS on the group-level reciprocity of the behaviours employed, we calculated the Kendall’s tau rowwise matrix correlation between the dyadic matrix and its transposed. Note, that proximity is by definition a reciprocal measure. Note further, that rowwise tau values could not be calculated for submissive behaviours (leave, submissive signal and avoidance), as these matrices were sparse. At increased LPS, the rowwise tau values of LIKE attitudes and affiliative behaviours increased (see black box-plots in Fig. 3), indicating enhanced group-level reciprocity. Similarly, at increased LPS the rowwise tau values of aggressive behaviours increased from negative values towards values around zero (see black box-plots in Fig. 3), indicating decreased group-level imbalance.

The increased reciprocity of affiliation at increased LPS can be explained as follows. In the null model setting (LPS = 0), individuals do not use LIKE attitudes to select affiliation partners. As grooming was directed up the hierarchy, LIKE attitudes were directed down the hierarchy, but had no feedback on affiliative behaviours. At increased LPS (LPS>0), individuals affiliated more selectively with individuals towards which they directed high LIKE attitudes. More frequent affiliation with these individuals reinforced the differentiation between preferred and non-preferred partners. Moreover, this feedback between affiliation and LIKE attitudes resulted in LIKE attitudes becoming more symmetric. At increased LPS (LPS>0), individuals also approached more selectively those individuals towards which they directed high LIKE attitudes. Therefore, proximity was increasingly determined by affiliation and LIKE attitudes and individuals were more often in proximity of similar-ranking individuals. This also allowed aggressive behaviours to become less imbalanced, as similar-ranked individuals also had more similar win chances than distant-ranked individuals.

### 2. Dynamic vs. fixed Attitude Model

**Fig 4:**
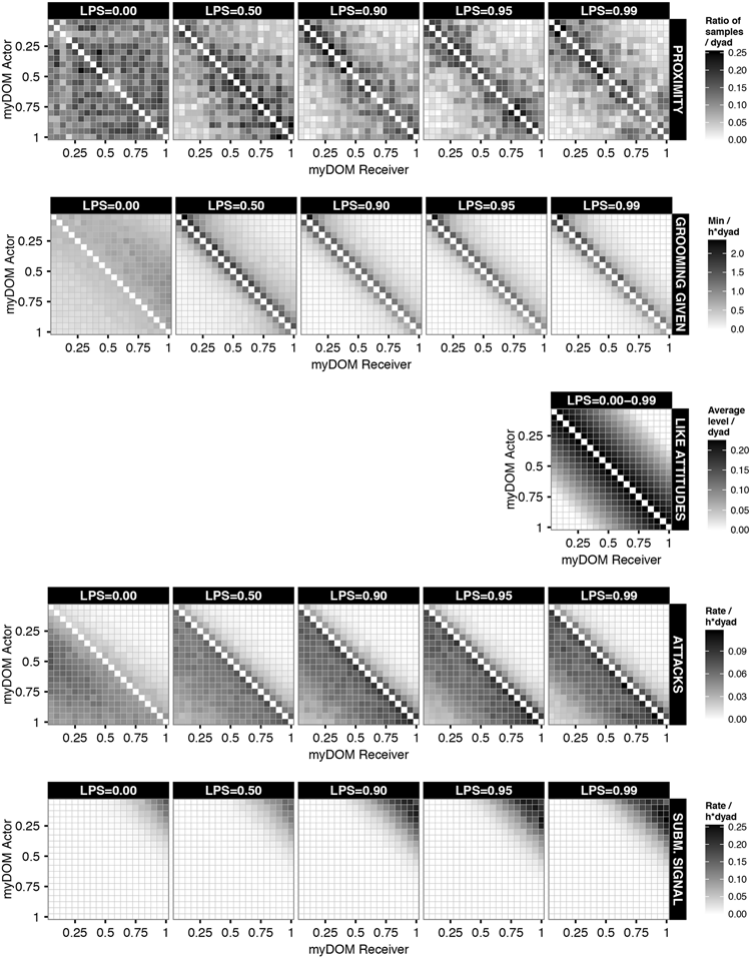
Interaction matrices of behaviours in the fixed attitude model. This figure shows the dyadic behavioural rates of a group at different settings of selectivity (LPS) in the fixed attitude model. For more details on the specific behaviours and how they were measured see caption of Fig. 2. The plot shows the behavioural rates of one example run averaged over one YEAR. Dark shades represent high rates or values. Values at the diagonal are by definition not applicable. Affiliative signals and approach are distributed very similarly to grooming given, aggressive signals are distributed very similarly to attacks, avoidance and leaving are distributed very similarly to submissive signals; these patterns are presented in Figure F7 in S1 Supplementary Material.

Above we have shown that when LPS was increased in the dynamic attitude model, reciprocity and the preference for similar-ranking individuals were reinforced. Here, we compare these results to the behavioural patterns that emerged in the fixed attitude model at increased LPS. In the fixed attitude model, LIKE attitudes are fixed and simply inversely related to rank-distance, and thus symmetric, with lower rank-distances corresponding to higher LIKE attitudes (cf Fig. 4 middle row). Thus, in the fixed attitude model, higher LPS results in higher selectivity for similar-ranking individuals. Remember, that when LPS is 0 in the fixed attitude model, the model has exactly the same settings as LPS = 0 in the dynamic attitude model; this is the null model setting, where individuals do not use their LIKE attitudes.

The differences in emotional states, behavioural rates and proximity of subordinates and dominants, and how these changed at increased LPS were very similar for the dynamic and the fixed attitude model (Figures F4 and F5 in S1 Supplementary Material).

In both models, the dynamic and the fixed attitude model, slight differences in behavioural rates towards similar-ranking and distant-ranking group members were already present in the null model (LPS = 0), and got generally reinforced at increased LPS (compare Fig. 1 and Figure F6 in S1 Supplementary Material). Of course, in the fixed attitudes model, LIKE attitudes do not change at increased LPS as they are by definition the same for all LPS.

In both models, at increased LPS the Shannon index decreased for agonistic and especially affiliative behaviours, and slightly increased for submissive behaviours (Figure F2 in S1 Supplementary Material), i.e. individuals restricted affiliative and aggressive behaviours to fewer group members and directed submission to slightly more group members. Also, at increased LPS affiliative behaviours were more reciprocated and aggressive behaviours were less imbalanced in both models (Fig. 3). Note, however, that in the fixed attitude model a low Shannon index and high reciprocity were already apparent at intermediate LPS (LPS> = 0.5), whereas this only emerged at high LPS (LPS = 0.99) in the dynamic attitude model (see below).

A clear difference between the dynamic and fixed attitude model was found in the dyadic distribution of some behavioural patterns. In the fixed attitude model, affiliative behaviours (grooming, affiliative signaling and approach) were more focussed on a few very similar-ranking partners, usually ranking just above or below ego (Fig. 4 and F7 in S1 Supplementary Material), while partners were more diverse in the dynamic attitude model (Fig. 2 and F3 in S1 Supplementary Material). Moreover, a strong preference for similar-ranking affiliation partners was already apparent at intermediate LPS (LPS> = 0.5) in the fixed attitude model (Fig. 4 and F7 in S1 Supplementary Material), whereas this only emerged at high LPS (LPS = 0.99) in the dynamic attitude model (Fig. 2 and F3 in S1 Supplementary Material). This was due to the fact that LIKE attitudes in the dynamic attitude model changed dynamically over time and needed to be maintained regularly, which is difficult at low LPS. In contrast, in the fixed attitude model, LIKE attitudes were fixed and did not need to be maintained. Here, individuals always prefer the same group members most, namely those that are most LIKEd, which are by definition most similar in rank. Hence, with LIKE attitudes being fixed, their effect on affiliative partner choice was stronger and already apparent at lower LPS compared to the dynamic attitude model, which was also reflected in the Shannon index (Figure F2 in S1 Supplementary Material) and reciprocity (Fig. 3).

Moreover, the dynamic and fixed attitude model differed in the partner-specificity of some behavioural patterns. In the fixed attitude model, affiliative partner choice is purely based on rank-distance (via fixed LIKE attitudes that are based on rank distance) and as a result behavioural rates within the dyads of the same rank-distance showed low variation. In contrast, in the dynamic attitude model affiliative partner choice is dependent on the specific identity of actors of earlier received affiliation. As a result, affiliative relationships were highly individualized and behavioural rates within dyads of the same rank-distance showed high variation. To quantify this, we calculated the standard deviation (SD) over the behavioural rates for all dyads of a certain rank-distance and then averaged the SD over all rank-distances.

**Fig 5:**
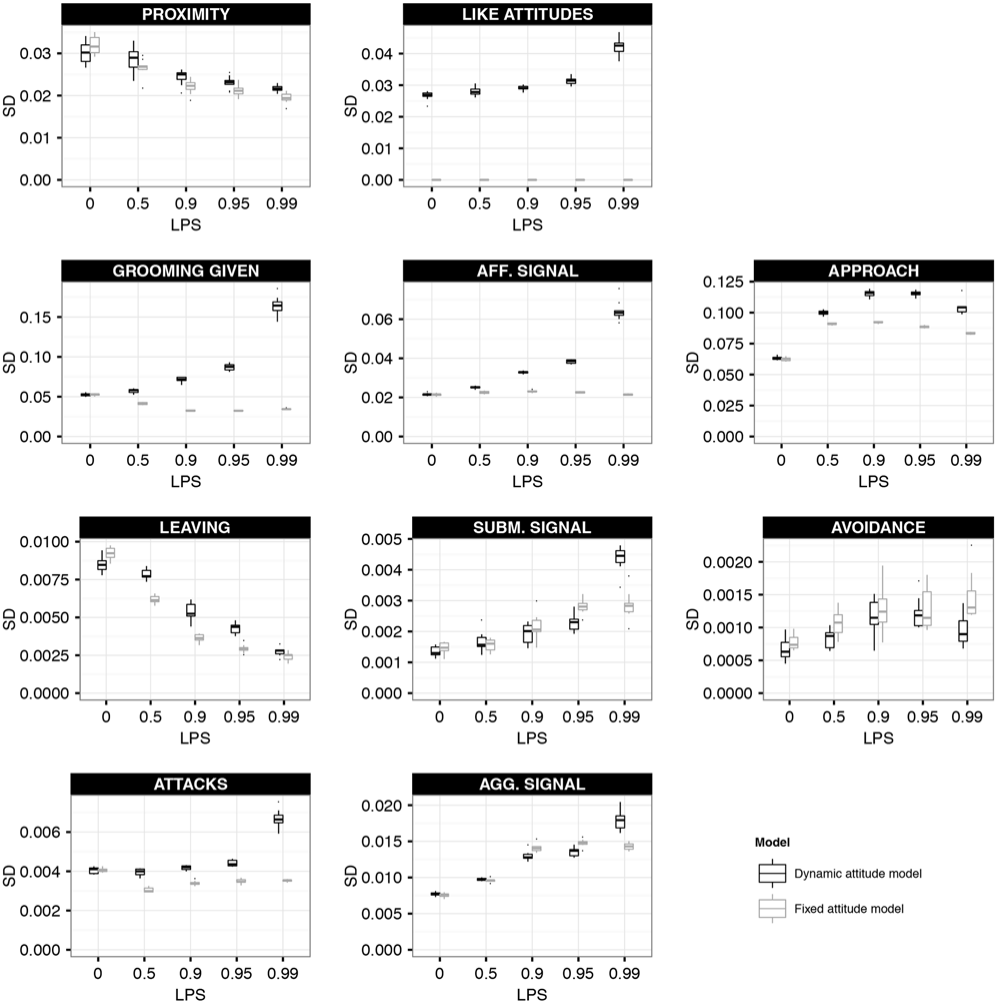
Partner-specificity of behaviours. This figure shows the standard deviation (SD) over the behavioural rates of dyads with the same rank-distance in the dynamic attitude model (black boxplots) and the fixed attitude model (grey boxplots). SD was calculated over all dyads of the same rank-distance based on the behavioural matrices averaged over one YEAR, and then averaged over all rank-distances. The box-plots show the results of 10 simulation runs. Note that LIKE attitudes in the fixed-attitude model are strictly based on rank-distance and therefore SD across dyads of same rank-distance is by definition zero.

At LPS = 0, SD was similar for both models (except of course for the differently defined LIKE attitudes), as here individuals in both models had no preference for specific affiliative partners (Fig. 5). At increased LPS, the SD of LIKE attitudes, affiliative behaviours (especially grooming) and attacks increased in the dynamic attitude model, while they increased not at all or to a lesser degree in the fixed attitude model (Fig. 5). Thus, in the dynamic attitude model, dyads of the same rank-distance showed more variation in their LIKE attitudes, the rates of affiliation and in the rates of attack than in the fixed attitude model. This pattern is also apparent from the matrix plots (compare Fig. 2 with 4 and Figure F3 with F7 in S1 Supplementary Material). For instance, when comparing the matrix of grooming given at LPS = 0.99 in both models (Figs. 2 and 4), dyads with rank-distance of 1, i.e. dyads just above or below the diagonal, varied less in their grooming rate in the fixed than in the dynamic attitude model. This shows that affiliation, attacks and LIKE attitudes in the dynamic attitude model were not purely influenced by rank-distance, but were also dependent on the individual-specific history of received affiliation, especially at high LPS.

At increased LPS, the SD of proximity scores and leaving rates decreased and that of agonistic signals and avoidance increased similarly for both models (Fig. 5). This suggests that the distribution of these behaviours was in both models mostly dependent on rank-distance. Moreover, it is interesting to note that affiliative patterns of our model are not purely a result of spatial group patterns.

### 3. Predictability within the Dynamic Attitude Model

**Fig 6:**
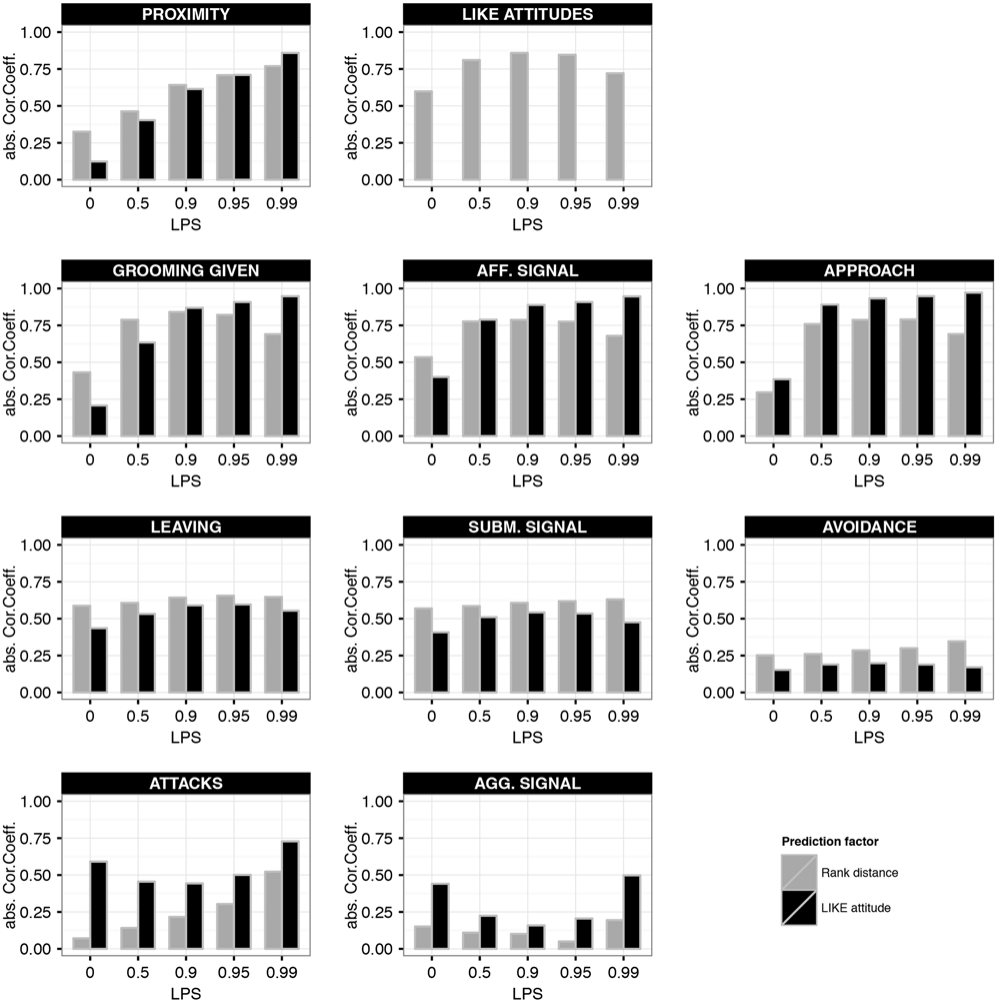
Correlation between behaviours and LIKE or rank distance in the dynamic attitude model. This figure shows the absolute row-wise Pearson correlation coefficients between the behavioural patterns and two predictive factors: Rank distance (grey) and LIKE attitudes (black). We used matrices of the behaviours and LIKE attitudes averaged over one YEAR. The correlation coefficient was calculated for each of 10 simulation runs and then averaged using a Fisher-z transformation.

Above we showed that high selectivity for affiliative partners (LPS) is necessary for the emergence of enhanced reciprocity and preference of similar-ranking affiliation partners within the group. Moreover, at high LPS only dynamic and not fixed LIKE attitudes allow for individual-specific relationships, in which behavioural rates vary across dyads of the same rank-distance. This raises the question how well the affiliative relationships in the dynamic attitude model are predicted by either rank-distance or LIKE attitudes and how this predictability depends on LPS. To investigate this, we calculated the row-wise correlation coefficients between the behavioural patterns and, respectively, LIKE attitudes or rank-distances (Fig. 6).

First, note that the correlation between the dynamic LIKE attitudes and absolute rank-distance first increased at increased LPS (LPS<0.9), but decreased again at even higher LPS (LPS>0.9) (see second panel of first row in Fig. 6). This means that LIKE attitudes corresponded well to rank-distance at intermediate LPS, but less at low or high LPS. At low LPS, LIKE attitudes were mainly directed down the hierarchy (Fig. 4). Therefore, LIKE attitudes were less well correlated to the symmetric rank-distance. On the other hand, at high LPS, LIKE attitudes were more based on individual-specific affiliative histories and therefore less derivable from rank-distance.

Proximity, grooming and affiliative signals were slightly better predicted by absolute rank-distance than by LIKE attitudes at lower LPS (LPS< = 0.5), however at high LPS (LPS> = 0.95) this was the other way around. Approach was generally best predicted by LIKE attitudes, especially at high LPS (LPS = 0.95).

Aggressive behaviours were best predicted by LIKE attitudes, especially at high or low LPS. This can be explained as follows. At low LPS, both LIKE attitudes and aggression are directed down the hierarchy, while absolute rank-distance is symmetric. At high LPS, LIKE attitudes and attacks become more reciprocated and symmetric, but also more individual-specific. Only submissive behaviours were always better predicted by rank-distance independently of LPS. This was due to the fact that submissive behaviours were not affected by LIKE attitudes or resulting proximity, as they were mostly directed towards group members distant in rank, which were also often distant in space.

To summarize, LIKE attitudes were best predictors of proximity, affiliative and aggressive behaviours, given that affiliative partner preference was high enough (LPS>0.9). At lower LPS, rank-distance corresponded better to grooming, lip-smacking and proximity and would, therefore, be a better cue to use when selecting affiliation partners.

## Discussion

### Emotional bookkeeping versus fixed attitude model

In the dynamic attitude model, which models emotional bookkeeping, only very high LIKE partner selectivity (LPS = 0.99) resulted in enhanced partner-specific reciprocity of both LIKE attitudes and grooming. This emerged via the following feedback loop: grooming increases the LIKE attitude towards the groomer, in turn this high LIKE attitude results in grooming of this LIKEd partner, but only when LIKE partner selectivity (LPS) is high enough. This shows that emotional bookkeeping can mediate partner-specific reciprocal patterns in LIKE-attitudes and grooming. Rank distance predicted LIKE attitudes and grooming across a broad range of LPS; only with very strong partner selectivity grooming was predicted better by LIKE attitudes than by rank distance (Fig. 6) and did reciprocal affiliation become partner-specific.

In the fixed attitude model, which is a symmetry-based model and lacks emotional bookkeeping, the LIKE attitude towards an individual depends merely on the rank difference with that individual; similar-ranked dyads were given a higher LIKE value than distant-ranked ones. So, here, by definition the LIKE attitudes are symmetrical and inversely related to rank distance. This model generated many patterns that were similar to the dynamic attitude model. This concerned general patterns of rates and direction of aggression, submission and affiliation. The average levels of arousal and emotional states, anxiety and satisfaction were quite similar as well. Similarly to the dynamic attitude model, affiliative patterns were more reciprocal at higher settings of LPS, thus when (fixed) LIKE-attitudes had a stronger effect on partner selection. However, the two models differ in two aspects. First, in the fixed attitude model the emerging grooming patterns showed high rates only for adjacently ranked individuals, whereas in the dynamic LIKE attitude model preferred partners consisted of individuals within a broader range of rank distances, which is what we see in real primate groups [30,32]. Second, in the fixed attitude model there is no partner-specific variation in affiliation within subgroups of dyads having a similar rank-distance, whereas reciprocal partner-specific LIKE attitudes and corresponding grooming exchanges are clearly present in the dynamic LIKE attitude model. The role of dynamic FEAR attitudes on the dynamics of LIKE attitudes remains to be explored.

This comparison of the two models generates new predictions: when individual recognition and emotional bookkeeping are present, reciprocal partner-specific affiliation may emerge and partner-specific emotional attitudes are better predictors of affiliative relationships than similarity characteristics of dyads, such as in this case rank distance. More speculatively, a system with dynamic relationships based on emotional bookkeeping is expected to be less vulnerable to cheaters than a system where affiliative partner choice is determined by a (fixed) similarity characteristic. In this light, the emotional bookkeeping model serves as a promising candidate model for the development and maintenance of affiliative relations in many primate species.

### Determinants of the behavioural patterns in the dynamic attitude model

Although partner-specific affiliation was an important feature in the dynamic LIKE attitude model, both rank distance and LIKE attitude were associated with patterns in behaviour. We determined which of the two better predicted these patterns. LIKE attitudes were the best predictors of proximity, affiliative and aggressive behaviours, given that affiliative partner selectivity was high enough (LPS> = 0.95). At lower LPS, rank-distance corresponded better to grooming, lip-smacking and proximity and would, therefore, be a better (and cognitively simpler) cue to use when selecting affiliation partners. Thus, the dynamic LIKE attitude model does not exclude dyadic similarity effects, but adds additional variation resulting in partner-specific relationships.

The outcomes of the EMO-model are based on a group size of 20 individuals. This group size lies within the range of group sizes of many primate species [78–82], amongst which many macaque species [83–87]. We expect that similar results will be found in a large range of group sizes, since differentiation in partner-specific affiliation will usually be found. However, future model and empirical studies are required to address the effect of group size.

That LIKE attitude was a better predictor of attacks than rank distance is interesting and not straight forward. At low partner selectivity, dominants were groomed often by subordinates. This resulted in LIKE attitudes directed down the hierarchy, which coincides with the pattern of aggression. On the other hand, at high partner selectivity, reciprocal LIKE attitudes developed mainly between similar-ranking individuals. Here, LIKE attitudes coincided with the bidirectional pattern of aggression, which was a result of smaller rank distances and was additionally mediated by frequent proximity of similar-ranking individuals. Empirical data show similar patterns of high rates of both grooming and aggression among individuals with close relationships, such as kin (grooming: e.g. [32,33]; aggression: [88]).

Recently, two other agent-based models of reciprocity have been published: the GrooFi model [41] and its extension FriendsWorld [89]; and the multi-generational model of Campenni and Schino, further called the C&S model [55]. We compare these ABMs to the EMO-model. The GrooFi model also generates reciprocal patterns in grooming [41]. Besides the obvious precondition of grouping allowing repeated interactions, the GrooFi model shows that none of the three cognitive capacities (individual recognition, memorizing earlier or recent affiliative interactions and partner selectivity) is necessary for reciprocity. The entities in this model do not recognise or remember each other (they only perceive each others dominance rank). ‘Choosing’ whom to groom occurs randomly from the animals that happen to be nearby. Thus, this model shows that group living entities which lack the three mentioned cognitive capacities can indeed show a correlation between giving and receiving of grooming at a group level. However, whether the GrooFi model represents reciprocity based on dyadic similarity or partner-specific preferences is not presented. Given the implementation of the model, it is likely that proximity determines reciprocity; indeed, the Tau-Kr correlation of grooming reciprocation at a group level in the full GrooFi model was 0.31 and 0.45 (depending on the intensity of aggression set in the model), which became-0.52 and 0.00, when the spatial structure was taken out of the model (tables 4 and 5 in [41]). The spatial proximity pattern in the GrooFi model is caused by the aggressive interactions and therefore reflecting the rank distances [41–43]. In contrast, in the EMO-model the proximity pattern is caused by agonistic interactions (involving fleeing upon being attacked, leaving or avoiding a potential aggressor) regulating repulsive movements among the model entities *as well as* by the approach behaviour steered by the LIKE attitudes, regulating attractive movements. Both rank distance and LIKE attitude were about equally predictive of proximity.

In a modified version of the GrooFiWorld model, FriendsWorld [89], group members with whom an individual engages most in grooming (top quartile) have been defined as "friends" and model individuals preferably approach such "friends". However, preference for grooming or grooming partner choice is not affected by these "friendships". Similarly to the EMO-model, preferred proximity to "friends" in FriendsWorld shapes the spatial structure of the group and in this way reinforces grooming reciprocity. Whether this model yields partner-specific grooming reciprocity or whether reciprocity is derivable from dominance relations is not presented. Individuals in the FriendsWorld model may employ a simple form of emotional bookkeeping to distinguish "friends" from "non-friends". However, the underlying (cognitive) mechanism is not explicitly formulated in the model and "friends" are determined by the global observer, i.e. by calculating for each individual the top quartile of its grooming partners. It is questionable whether it is cognitively less demanding for an individual to keep track of the exact amount of grooming with all group members (over the whole timeframe of the simulation) which is necessary to determine its "friends" at any moment, compared to the emotional bookkeeping of LIKE attitudes implemented in the EMO-model.

The C&S model [55] does not include a spatial environment in which the entities move around and can encounter or avoid each other. The single behaviour included in this model is directing a cooperative act towards another agent. In contrast, individuals in the EMO-model have a broad repertoire of behaviours. The authors focus on the explanatory factor ‘partner choice based on benefits received’ and show that the degree of reciprocation of cooperative acts depends on the time span of memory of earlier received cooperative acts: with longer memory time span a stronger degree of reciprocation results. Lacking any spatial environment, this ABM could not address the question of the relative impact of spatial pattern and memory on the emergence of reciprocation in the group. In contrast, the GrooFiWorld and FriendsWorld models, in which partner choice based on benefits received is lacking, show that the spatial proximity pattern has a strong influence on the emergence of reciprocation at a group level. Our EMO-model shows that added to this effect of proximity pattern on reciprocity in the group, *partner-specific* reciprocal relationships arise when the model agents are very selective in their partner choice. This last factor, degree of partner selectivity, is not included in either the C&S model or in the GrooFi- or FriendsWorld, so our and their results cannot be compared in this respect. In the C&S model two factors, namely memory time span and size of the subset of candidates, are systematically varied. This last factor concerns the number of candidates (N = 2, 10, 25 or 49) which are randomly extracted from the population, and from which the actor selects as receiver of its cooperative act, the one that has been most cooperative towards him. So, whereas in the GrooFiWorld, FriendsWorld and EMO-model the subset of candidates to choose from is determined by spatial proximity, in the C&S model the candidates are at each step randomly selected from the whole population, and can thus vary greatly from step to step. The factor ‘memory time span’ will be the topic of a future paper in which the EMO-model is used to address the question of stability of reciprocal grooming partner preferences.

The current EMO-model is a “single-generation” model focusing on the proximate mechanisms that lead to reciprocity. For future research, it would be interesting to include evolutionary processes in the EMO-model. This has been done in the “multi-generation” C&S model [55]. Here, individuals employ selfish or cooperative strategies, where cooperation implies a fitness cost for the actor and a benefit for the receiver. When the benefits of receiving cooperation were high enough relative to the costs of giving cooperative acts, agents with a cooperative strategy were evolutionarily successful. It remains to be investigated whether in an evolutionary version of the EMO-model a similar result will be found. In the model without any partner selectivity (LPS = 0 for all individuals) evolutionarily stable reciprocity may not arise because selfish agents will continue to be ‘selected’ by others as receivers of grooming due to spatial proximity, since they will receive benefits without incurring any costs. However, when in the dynamic attitude model the strength of partner selectivity varies between individuals, selfish agents will be chosen less as receivers of grooming. Therefore, in such a model, which includes partner choice based on benefits received, evolutionarily stable reciprocity may be found. In these evolutionary versions of the EMO-model the evolutionary success of individuals that differ in strength of partner selectivity or length of timeframe of emotional bookkeeping may be investigated. The different effects of spatial self-organization and selection of fitness-related behavior on the evolutionary success of different behavioral strategies may be explored in these models.

### Cognitive processes underlying reciprocity

Reciprocity of affiliative behaviour has been found in primates. In particular grooming is often exchanged for grooming. That reciprocity is found is no longer surprising and the current discussion focuses on the cognitive processes that determine this reciprocity, ranging from simple to complex (see Introduction). In real primates, it is difficult to disentangle which cognitive process underlies reciprocity. When simpler factors, such as rank distance or proximity, can explain (a certain degree of) reciprocity, these are preferred over more complex ones.

The current study explicitly implemented different cognitive processes into model entities. Both the fixed and dynamic attitude model generate reciprocity in affiliation. However, they differ when high partner selectivity determines partner choice. The fixed attitude model results in a small number of dyads with reciprocal affiliation, all similar in characteristics and strength of the affiliating dyad. In contrast, the emotional bookkeeping model generates a broad range of dyads, where affiliation is not purely based on rank-distance and where reciprocity is thus partner-specific. In primates, partner-specific affiliative relationships, i.e. social bonds or friendships, are found that cannot be explained solely on the basis of similarity in rank, kinship or age [12,25,32]. Moreover, primates recognize their group members (vocalisations: [90,91]; pictures: [92]; cross-modal recognition: [93]). This suggests that the partner-specific affiliative behaviour resulting from emotional bookkeeping may occur, possibly in combination with some dyadic similarity characteristic, but clear empirical evidence is lacking.

Several suggestions have been made how the process of emotional bookkeeping may be addressed empirically [50,94]. Although it is difficult to study ‘LIKE attitudes’ in primates directly, one can study the development of newly started affiliative relationships by scoring the time course of affiliative behaviours, from which the corresponding temporal pattern of LIKE attitudes of both A and B can be inferred. Two studies, both addressing the mechanisms of reciprocity in primates [50,94], make similar suggestions. In addition, new empirical evidence suggests how updating of ‘LIKE attitudes’ may take place: grooming with favoured partners may strengthen the bond by increasing oxytocin levels, while grooming with non-favoured partners does not have such an effect (chimpanzees: [10]; see also [9]). Therefore, primates may start and maintain relationships based on affiliative interactions, consistent with the emotional bookkeeping hypothesis.

Besides studying the time course of affiliative behaviour in specific dyads in a group, another suggestion for future research concerns the role of partner choice. In our dynamic LIKE attitude model the LPS parameter controls the degree of selectivity to choose a LIKEd partner. In line with Tiddi et al. [50], our modelling study also suggests to investigate the combined role of partner choice and temporal pattern of affiliative events in the emergence of reciprocity. More particularly, since in our model only very high values of LPS result in partner-specific reciprocal relationships, the question arises whether this strong selectivity of grooming partners also occurs in primate groups, or whether some form of LIKE updating is used by primates, differing from the one implemented in our model.

Partner selectivity can be promoted through spatial proximity. In turn, proximity can be promoted through partner selectivity, as highly LIKEd partners are approached more often than less LIKEd ones. Preferential engagement with partners, either based on fixed characteristics, such as rank-distance or kinship, or based on dynamic LIKE attitudes, can generate group patterns of social behaviour. This suggests that individual recognition may be an important organizing principle of social behaviour that does not necessarily require spatial differentiation, although the social interaction patterns in the group may be enhanced by spatial differentiation. Indeed, in experimental set-ups of providing benefits to others (i.e., pro-social behaviour) that control for spatial distance, individuals behave differently to individuals with whom they have different relationships: they differentiate between close social partners and other individuals and on the basis of relative dominance position [95,96]. Close associates and lower-ranking individuals are more often benefited than random group members or higher-ranking individuals [96]. This indicates that primates possess partner-specific attitudes and use these in their behavioural decisions.

In sum, there are clear indications that primates may use emotional bookkeeping to establish and maintain relationships. They have individual recognition, exert partner choice and have partner-specific attitudes. This does not preclude effects of rank-distance or other dyadic similarity characteristics, but may be formed on top of them. However, convincing empirical evidence of emotional bookkeeping is still lacking.

### Conclusion

In sum, reciprocity of grooming may depend on a simple rule involving symmetry-based behaviour, as modelled in the fixed attitude model, where FEAR and LIKE attitudes are both fixed and purely dependent on rank distance, and where individual recognition does not play a role. However, this model fails to generate partner-specific reciprocal affiliative dyads, and therefore cannot explain such patterns found in primates (e.g. [25]). In the dynamic attitude model partner-specific reciprocity patterns emerge due to emotional bookkeeping, which does require individual recognition. Here, two emotional dimensions regulate and are regulated by social behaviour, allowing for self-reinforcing feedback loops between behaviour selection and partner preferences. Embedding behavioural decision making into a framework of emotional processes promises to provide a better understanding of the links between different levels of organisation (e.g. [97]). Thus, using an ABM to model relatively complex cognitive processes can inform us on the behavioural consequences of these capacities relative to simpler processes. Our EMO-model suggests that emotional bookkeeping in combination with a high degree of partner selectivity (“choosiness”) may result in partner-specific affiliative relationships, suggesting that the more complex process of calculated reciprocity is not necessary for the emergence of differentiated social bonds.

## Supporting Information

S1 Supplementary Material This file contains figures F1-F7. (DOC)

S2 Supplementary Material This file contains tables T1-T3. (DOC)
