# Supplementary material for: Emotional Bookkeeping and High Partner Selectivity Are Necessary for the Emergence of Partner-Specific Reciprocal Affiliation in an Agent-Based Model of Primate Groups
Source: PLoS One. 2015 Mar 18;10(3):e0118921. doi: 10.1371/journal.pone.0118921 (PMC4364990; doi:10.1371/journal.pone.0118921)
Supplement: S1 Supplementary Material — (DOC) [file pone.0118921.s001.doc]

**SUPPLEMENTARY MATERIAL S1**


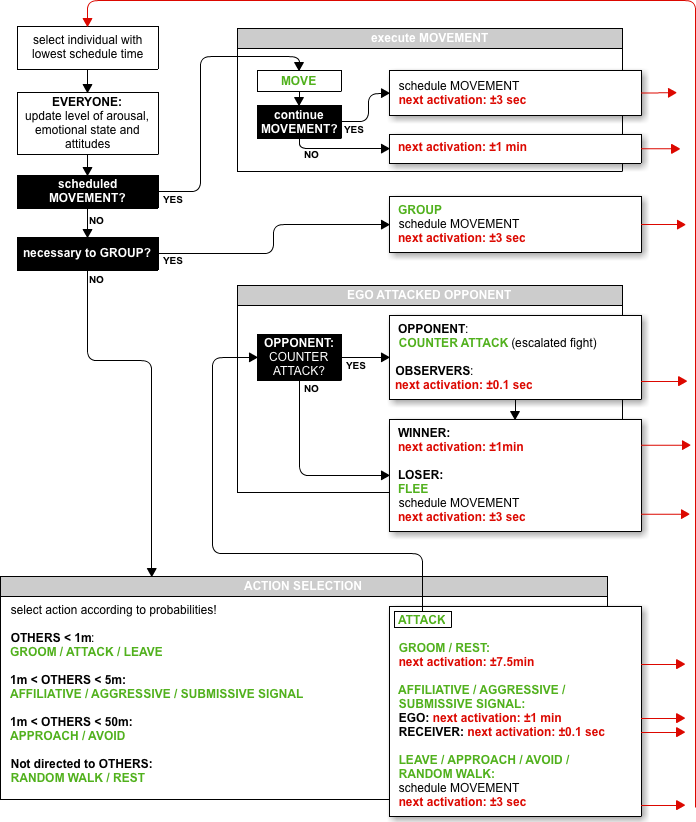


**Figure F1: Process overview of the model.** This figure illustrates the order of the processes executed by the model entities and their timing regime. From Evers E et al. (2014) The EMO-Model: An Agent-Based Model of Primate Social Behavior Regulated by Two Emotional Dimensions, Anxiety-FEAR and Satisfaction-LIKE. PLoS ONE 9(2): e87955. doi:10.1371/journal.pone.0087955.g001

**
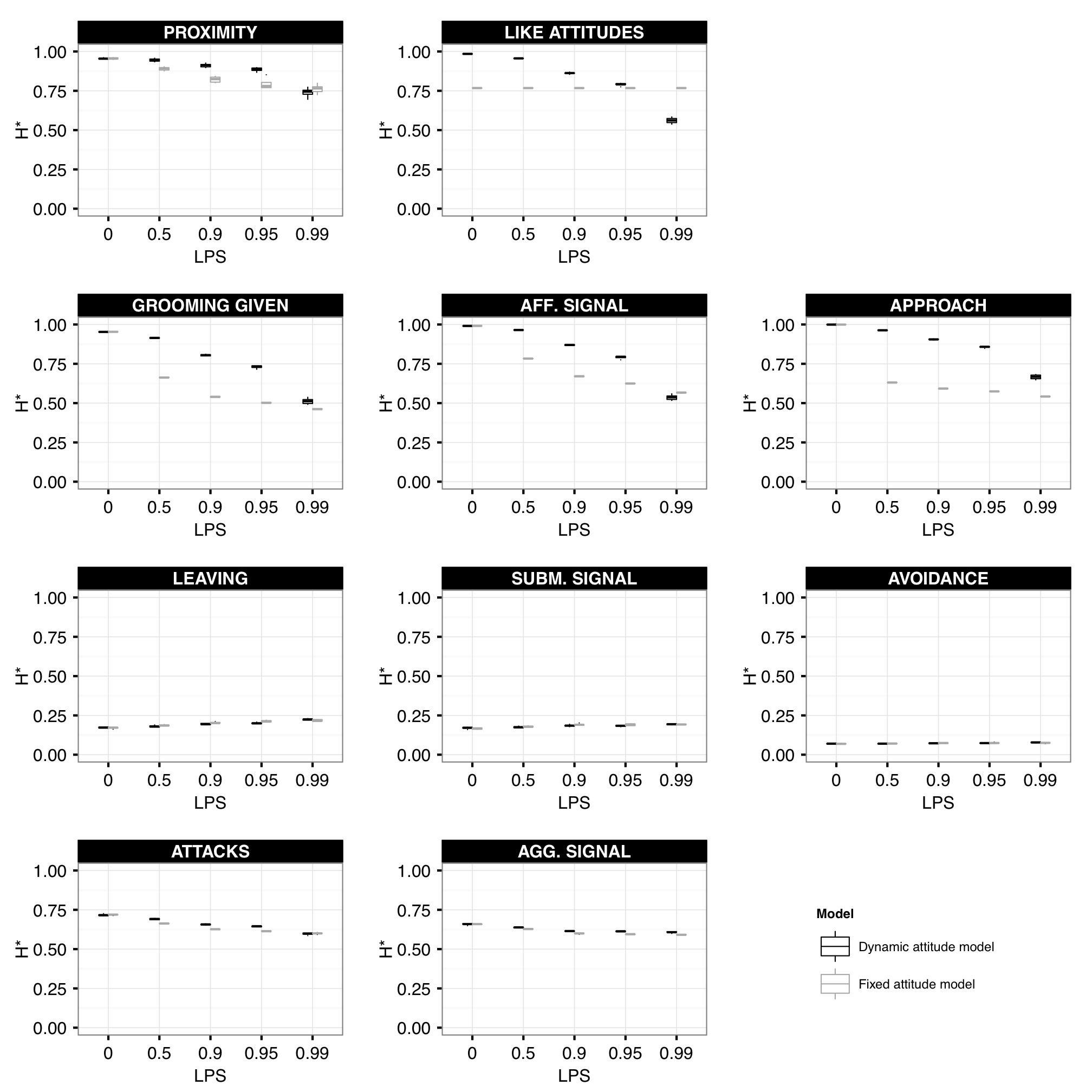
Figure F2: Shannon indices for the behaviours.**

This figure shows Shannon indices (H*) of the behaviours at different settings of selectivity (LPS) in the dynamic (black box-plots) and the fixed (grey box-plots) attitude model. A Shannon index of 1 indicates that the behaviour is distributed equally among all potential receivers, while a lower H* indicates that the behaviour is directed more selectively at certain individuals. H*=0 would indicate a behaviour that is only directed at one group member. Shannon indices were calculated based on the interaction matrices averaged over one year. The box-plots show the group means of 10 simulation runs.

**
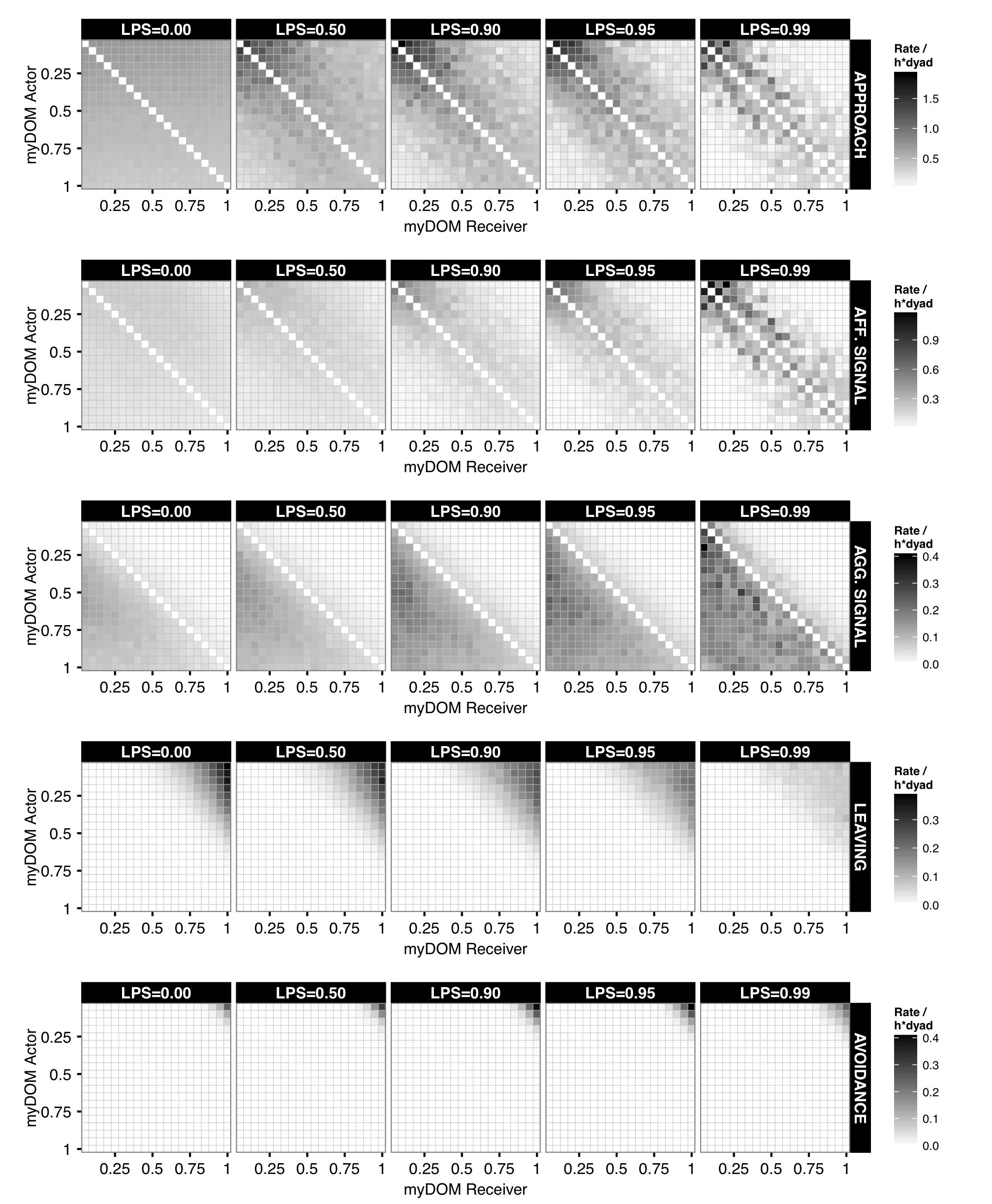
Figure F3**: **Interaction matrices of additional behaviours in the dynamic attitude model.**

This figure shows the dyadic behavioural rates of a group at different settings of selectivity (LPS) in the dynamic attitude model. Behaviours are directed from actors (y-axis) to receivers (x-axis), both are ordered by dominance strength, ranging from low-ranking (myDOM=0.05) to high-ranking (myDOM=1.0) individuals. All behaviours are measured in occurrences per HOUR. The plot shows the behavioural rates of one example run averaged over one YEAR. Dark shades represent high rates. Values at the diagonal are by definition not applicable. The distribution of proximity scores, LIKE attitudes, grooming given, attacks and submissive signals are presented in **Figure 2**.

**
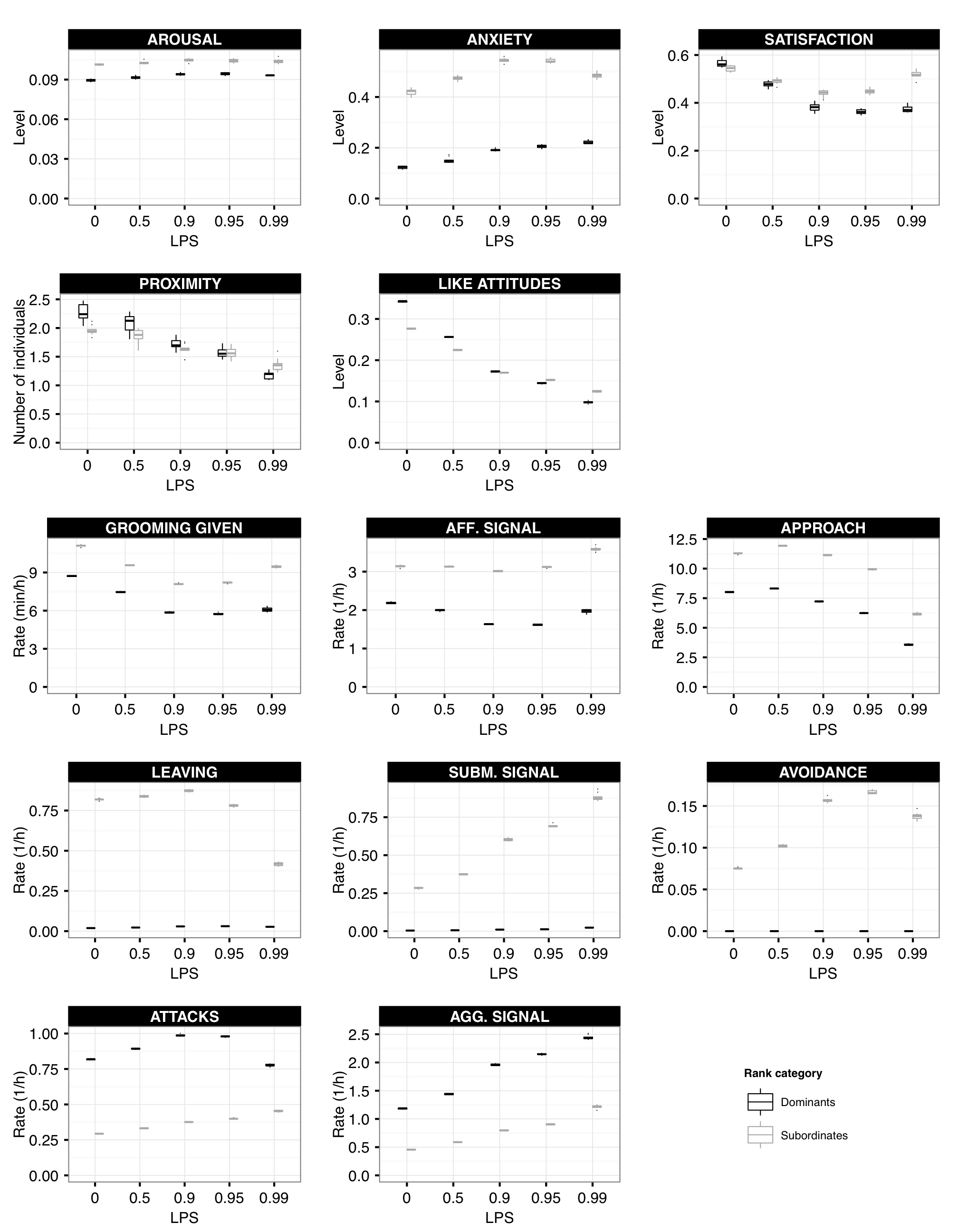
Figure F4**: **Emotional levels and behavioural rates per rank category in the dynamic attitude model.**

This figure shows the averaged levels of the emotional state and the rates of behaviour per individual for dominants (black box-plots) and subordinates (grey box-plots) at different settings of selectivity (LPS) in the dynamic attitude model. Levels of arousal, anxiety and satisfaction levels were averaged over one YEAR. For more details on the other measures see caption of **Figure 1**. The box-plots show the results of 10 simulation runs, averaged over 1 YEAR. From Evers E et al. (2014) The EMO-Model: An Agent-Based Model of Primate Social Behavior Regulated by Two Emotional Dimensions, Anxiety-FEAR and Satisfaction-LIKE. PLoS ONE 9(2): e87955. doi:10.1371/journal.pone.0087955.g004

**
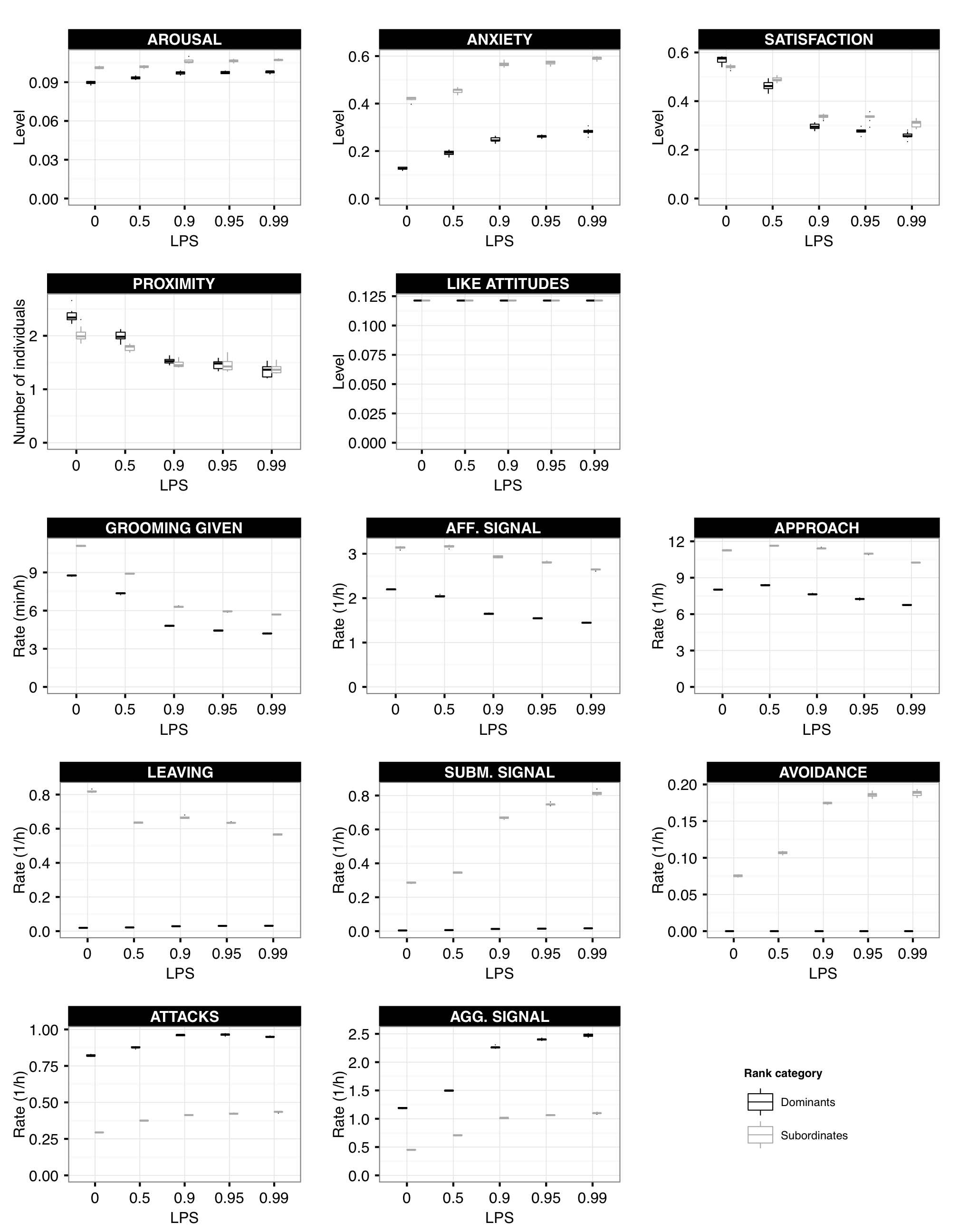
Figure F5**: **Emotional levels and behavioural rates per rank category in the fixed attitude model.**

This figure shows the averaged levels of the emotional state and the rates of behaviour per individual for dominants (black box-plots) and subordinates (grey box-plots) at different settings of selectivity (LPS) in the fixed attitude model. For more details on the measures see caption of **Figure 1** and **F4**. The box-plots show the results of 10 simulation runs, averaged over 1 YEAR.

**
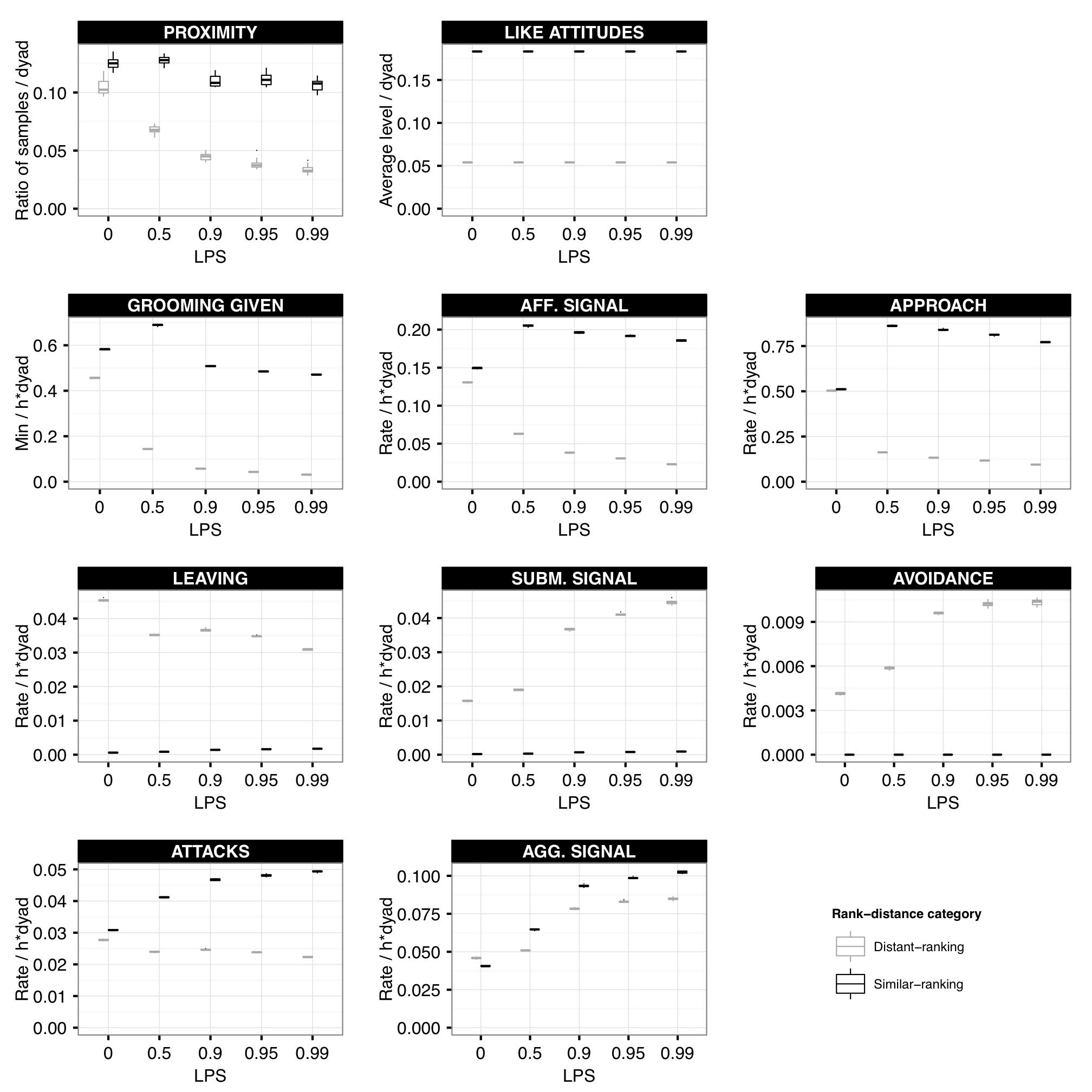
Figure F6**: **Behavioural rates per rank-distance category in the fixed attitude model.**

This figure shows the averaged behavioural rates for distant-ranked (grey box-plots) and similar-ranked (black box-plots) dyads at different settings of selectivity (LPS) in the fixed attitude model. For more details on the specific behaviours and how they were measured see caption of **Figure 1**. The box-plots show the results of 10 simulation runs, averaged over 1 YEAR.

**
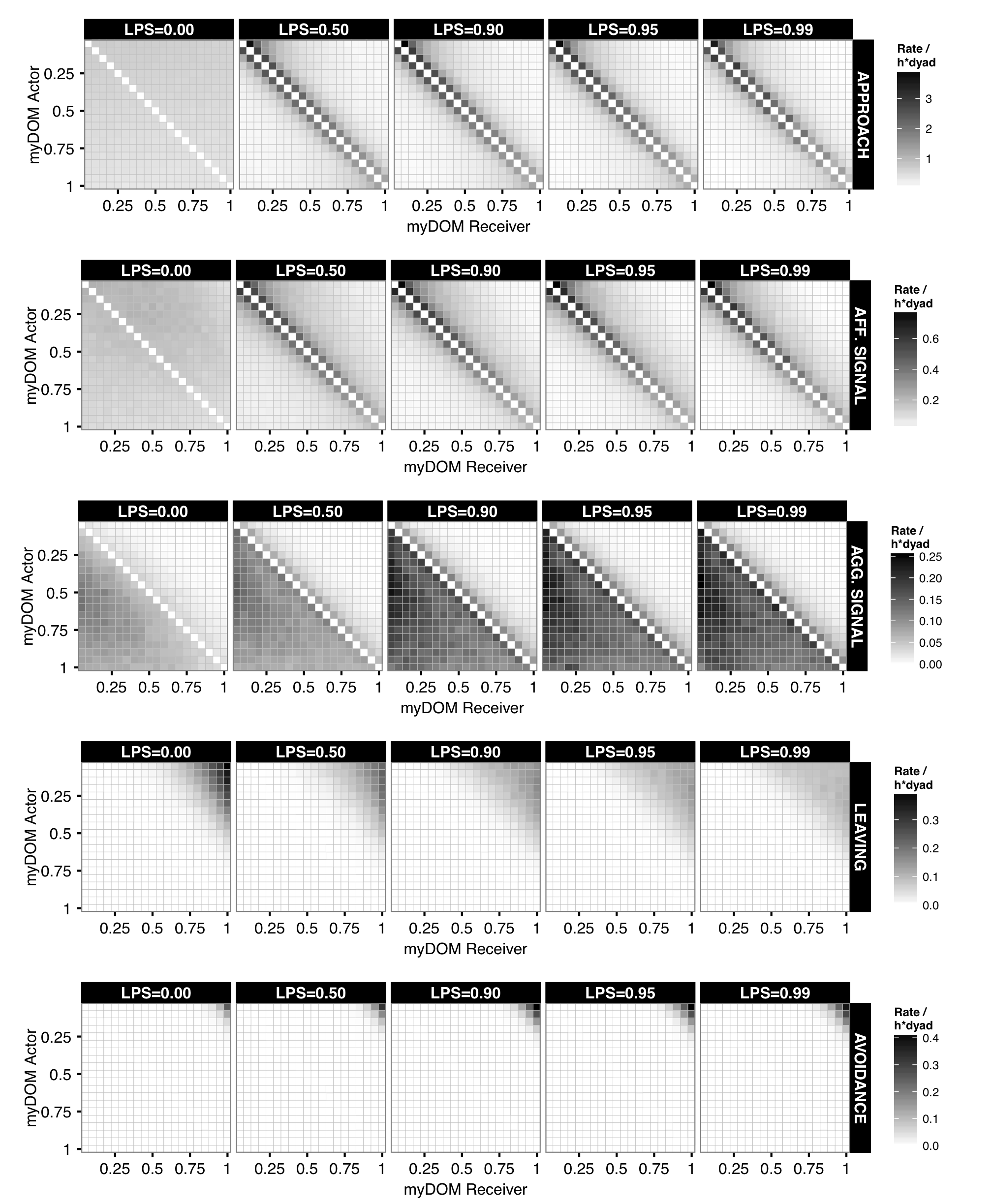
Figure F7**: **Interaction matrices of additional behaviours in the fixed attitude model.**

This figure shows the dyadic behavioural rates of a group at different settings of selectivity (LPS) in the fixed attitude model. Behaviours are directed from actors (y-axis) to receivers (x-axis), both are ordered by dominance strength, ranging from low-ranking (myDOM=0.05) to high-ranking (myDOM=1.0) individuals. All behaviours are measured in occurrences per HOUR. The plot shows the behavioural rates of one example run averaged over one YEAR. Dark shades represent high rates. Values at the diagonal are by definition not applicable. The distribution of proximity scores, LIKE attitudes, grooming given, attacks and submissive signals are presented in **Figure 4**.
