# Supplementary material for: Emotional Bookkeeping and High Partner Selectivity Are Necessary for the Emergence of Partner-Specific Reciprocal Affiliation in an Agent-Based Model of Primate Groups
Source: PLoS One. 2015 Mar 18;10(3):e0118921. doi: 10.1371/journal.pone.0118921 (PMC4364990; doi:10.1371/journal.pone.0118921)
Supplement: S2 Supplementary Material — (DOC) [file pone.0118921.s002.doc]

**Supplementary Material S2**

**Table T1: Individual-specific state variables of the model entities.**

| **State Variable Name** | **Description** | **(Initial) Value** | **Possible Range or Values** | **Fixed / Dynamic** |
| --- | --- | --- | --- | --- |
| **GENERAL STATE VARIABLES** | | | | |
| myTIME | Waiting time until next scheduled activation | 1 ± 0.05 min (mean ± SD) | May range between 0.1 ± 0.005 sec (fast reaction) and 7.5 ± 0.375 min (rest/groom) (depending on the social context) | Dynamic |
| myDOM | Dominance strength | between 1/N and 1.0 | Scaled between 1/N (lowest-ranking) and 1.0 (highest-ranking) | Fixed |
| mySCAN_PROB | Probability of employing scanning | depends on arousal and activity | May range between 0.0 and 1.0 (depending on arousal and activity) | Dynamic |
| myVIEW_ANGLE | Width of currently employed view angle | 120º | May be either 120º (not scanning) or 360º (scanning) | Dynamic |
| **EMOTIONAL STATE VARIABLES** | | | | |
| myAROUSAL | Arousal state | 0.09 | May range between 0.0 (relaxed) and 1.0 (aroused) | Dynamic |
| mySATISFACTION | Affiliation-related emotional state | 0 | May range between 0.0 (unsatisfied) and 1.0 (satisfied) | Dynamic |
| myANXIETY | Agonism-related emotional state | 0 | May range between 0 (not anxious) and 1 (anxious) | Dynamic |
| myAROUSAL_LIMIT | Arousal level that is approached over time | 0.09 | May be 0.03 (grooming received), 0.04 (grooming given), 0.09 (default), 0.12 (dominant perceived), 1.0 (maximum) | Dynamic |
| myANXIETY_LIMIT | Anxiety level that is approached over time | 0 | May be 0.0 (not anxious) or 1.0 (anxious) | Dynamic |
| mySATISFACTION_LIMIT | Satisfaction level that is approached over time | 0 | May be 0.0 (not satisfied) or 1.0 (satisfied) | Dynamic |
| **EMOTIONAL ATTITUDE VARIABLES** | | | | |
| FEAR_ij_ | Agonism-related emotional attitude from individual i to j | myDOM_j_ - myDOM_i_ | myDOM_j_ - myDOM_i_ | Fixed |
| LIKE_ij_ (dynamic attitude model) | Affiliation-related emotional attitude from individual i to j | 0 | May range between 0 (neutral) and 1 (preferred affiliation partner) | Dynamic |
| LIKE_ij_ (fixed attitude model) | Affiliation-related emotional attitude from individual i to j | 0.243 - abs(FEAR_ij_)*0.36 | 0.243 - abs(FEAR_ij_)*0.36 | Fixed |

**Table T2: General model parameters.**

| **Parameter** | **Description** | **Value** |
| --- | --- | --- |
| **General parameters** | | |
| N | Number of individuals in the group | 20 |
| D | Grid unit | 1 m |
| FIELD_SIZE | Field size | 300 x 300 m |
| MINUTE | Time step | 1 min |
| HOUR | 1 hour | 60 MINUTES |
| DAY | 1 day | 12 HOURS |
| WEEK | 1 week | 7 DAYS |
| YEAR | 1 year | 50 WEEKS |
| **Sensing parameters** | | |
| VIEW_ANGLE | Default view angle | 120º |
| MAX_ANGLE | View angle when scanning | 360º |
| FAR_DIST | Maximum tolerated distance to furthest group member | 100 m |
| MAX_DIST | Maximum distance to individually recognize group members | 50 m |
| NEAR_DIST | Maximum preferred distance to the group | 20 m |
| PERS_DIST | Maximum distance to perceive signals or escalated fights | 5 m |
| INTERACT_DIST | Maximum distance to physically interact with others | 1 m |
| MIN_OTHERS | Minimum preferred number of conspecifics in NEAR_DIST | 3 |
| **Movement parameters** | | |
| SPEED | Movement speed | 0.6 m/s |
| STOP_CHANCE | Probability of ending the current movement bout | 0.1 |

From Evers E et al. (2014) The EMO-Model: An Agent-Based Model of Primate Social Behavior Regulated by Two Emotional Dimensions, Anxiety-FEAR and Satisfaction-LIKE. PLoS ONE 9(2): e87955. doi:10.1371/journal.pone.0087955.s002

**Table T3: Effect of social behaviors on arousal, anxiety and satisfaction levels.**

| **Behavior** | **Change of arousal, anxiety or satisfaction level in the model** | **Parameter name** |
| --- | --- | --- |
| **Behaviors affecting arousal level** | | |
| Escalated fight observed | + 0.04 | EFO_AR_INC |
| Aggressive signal received | + 0.04 | ASR_AR_INC |
| Attack given | + 0.04 | AG_AR_INC |
| Attack received | + 0.08 | AR_AR_INC |
| Affiliative signal received | - 0.04 | AS_AR_DEC |
| Submissive signal received | - 0.04 | SS_AR_DEC |
| Default decrease | - 0.02 / min | DEF_AR_DEC |
| Default increase | + 0.02 / min | DEF_AR_INC |
| Proximity of dominant | + 0.02 / min | PD_AR_INC |
| Grooming given | - 0.02 / min | GG_AR_DEC |
| Grooming received | - 0.04 / min | GR_AR_DEC |
| **Behaviors affecting anxiety level** | | |
| Escalated fight observed | + 0.2 | EFO_ANX_INC |
| Aggressive signal received | + 0.2 | ASR_ANX_INC |
| Attack given | + 0.2 | AG_ANX_INC |
| Attack received | + 0.4 | AR_ANX_INC |
| (Escalated) fight lost | + 0.4 | EFL_ANX_INC |
| Affiliative signal received | - 0.2 | ASR_ANX_DEC |
| Submissive signal received | - 0.2 | SSR_ANX_DEC |
| (Escalated) fight won | - 0.4 | EFW_ANX_DEC |
| Default anxiety decrease rate | - 0.002/min | DEF_ANX_DEC |
| Anxiety decrease rate when giving grooming | - 0.01/min | GG_ANX_DEC |
| Anxiety decrease rate when receiving grooming | - 0.02/min | GR_ANX_DEC |
| **Behaviors affecting satisfaction level** | | |
| Satisfaction increase rate when giving grooming | + 0.05/min | GG_SAT_INC |
| Satisfaction increase rate when receiving grooming | + 0.10/min | GR_SAT_INC |
| Default satisfaction decrease rate | - 0.02/min | DEF_SAT_DEC |

In our model, we distinguished between point behaviours, which affect the level of arousal, anxiety or satisfaction instantly (e.g. 'attack received'), and duration behaviours or social contexts, for which the effect on the emotional state depends on the duration the behaviour or context is experienced (e.g. 'grooming received'). From Evers E et al. (2014) The EMO-Model: An Agent-Based Model of Primate Social Behavior Regulated by Two Emotional Dimensions, Anxiety-FEAR and Satisfaction-LIKE. PLoS ONE 9(2): e87955. doi:10.1371/journal.pone.0087955.s005
